# Chronic hypertension impairs lymphatic drainage in deep cervical lymph nodes

**DOI:** 10.1172/jci.insight.206714

**Published:** 2026-08-11

**Authors:** Kaiming Xu, Ankita Bhardwaj, Sunil Koundal, Qin Ren, Chenyu You, Xenophon Papademetris, Helene Benveniste, Tryphon T. Georgiou

**Affiliations:** 1 Department of Anesthesiology, Yale School of Medicine, New Haven, Connecticut, USA.; 2 Department of Computer Science and; 3 Department of Applied Mathematics & Statistics, Stony Brook University, Stony Brook, New York, USA.; 4 Departments of Biomedical Informatics & Data Science, Radiology & Biomedical Imaging, and Biomedical Engineering, and; 5 Yale Biomedical Imaging Institute, Yale School of Medicine, New Haven, Connecticut, USA.; 6 Department of Mechanical and Aerospace Engineering, University of California, Irvine, California, USA.

**Keywords:** Aging, Immunology, Hypertension

## Abstract

The glymphatic-meningeal pathway, important for brain homeostasis, depends on the drainage function of the cervical lymphatic system. Although new therapies aim to modulate this pathway, a lack of methods for quantifying lymphatic drainage function hinders our ability to understand how targeting the cervical lymph nodes may benefit brain health. To address this, we developed and applied a fluid transport model to dynamic contrast-enhanced MRI (DCE-MRI) data to visualize and quantify tracer-tagged lymph through the deep cervical lymph nodes (dcLNs). The model incorporated physical principles of solute transport to provide a biologically interpretable framework for analyzing microflows in real time. We applied this model to investigate the effects of chronic hypertension on dcLN drainage by comparing normotensive Wistar-Kyoto rats with spontaneously hypertensive stroke-prone (SHRSP) rats. In normal rats, the model revealed complex and tortuous lymph streams of a 200 kDa tracer transported through the sinus system of the dcLNs. In contrast, SHRSP rats exhibited significantly altered fluid dynamics, characterized by simpler stream patterns and reduced flow through the dcLNs. These findings demonstrated that untreated chronic hypertension adversely affects lymph node drainage function. This study provides potentially new insight into impaired lymphatic drainage as a mechanism linking systemic disease to brain health.

## Abstract 2

Using a novel computational MRI imaging framework, we show that chronic high blood pressure impairs lymph node drainage of brain-derived fluids, potentially contributing to cognitive decline and neurodegeneration.

## Introduction

The meningeal lymphatic vasculature and interconnected downstream cervical lymph nodes are essential for brain homeostasis, by draining excess fluid and waste-laden cerebrospinal fluid (CSF) and interstitial fluid (ISF) from the brain (1–5). Impairment of this brain clearance pathway is associated with several pathologies including cognitive dysfunction (2, 6), blood-brain barrier disruption (3), and neurodegeneration (2). Significant advances in MRI have enabled noninvasive visualization of glymphatic (7–10) and meningeal lymphatic vasculature transport (11, 12). Yet, significant scientific challenges remain in evaluating the functional drainage capacity of cervical lymph nodes — a bottleneck with direct clinical consequence. Emerging interventions, including noninvasive pharmacological approaches (13, 14) and the recently proposed cervical lymphovenous bypass surgery for neurodegenerative diseases such as Alzheimer’s disease (15), depend critically on the ability to assess brain-to-cervical lymph node drainage in functional terms. Although current methods, including intrathecal gadolinium contrast in combination with T1 mapping by MRI, can trace CSF-ISF outflow to the cervical nodes in rodents (16) and in humans (17), they have so far provided limited information on intranodal drainage function. Therefore, developing a robust method for quantifying nodal lymph flow in vivo remains an urgent priority for the field.

To meet this challenge, we adapted a foundational theoretical fluid dynamics model, applying its principles to the study of brain-to-cervical lymphatic drainage. Specifically, we adapted the Schrödinger Bridge model, originating in Erwin Schrödinger’s work in the early 1930s (18, 19), aimed at estimating particle trajectories between (i.e., bridging) data at the start and end of an observation period. Briefly, from a statistical perspective, the Schrödinger model computes a posterior probabilistic model of aggregate flow by minimizing its relative entropy (Kullback-Leibler divergence) from a prior model, thereby identifying the most likely flow lines consistent with the observed data; the prior model incorporates general system information (e.g., biological information), whereas the posterior model is the updated model that best explains the data. We refer to this specific computation for visualizing tracer-tagged lymph flow as the generalized Schrödinger Bridge (gSB) model.

To examine the performance and sensitivity of the gSB model in detecting abnormalities in lymphatic flow, we applied it to map deep cervical lymph node (dcLN) lymph flow of normal and chronic hypertensive rats using the spontaneously hypertensive stroke-prone (SHRSP) rat model (20, 21). Specifically, SHRSP rats with chronic hypertension have an inherent predisposition to develop small vessel cerebral disease and exhibit cerebral pathology similar to those in humans, including thickening of the cerebral arterioles (arteriolosclerosis), lacunar stroke (22), enlarged perivascular spaces (23), and blood-brain barrier dysfunction (24). Clinically, it has been well-documented that cerebral small vessel disease is associated with cognitive decline and dementia (25). Notably, preclinical studies have shown that glymphatic transport is reduced in SHRSP rats (26, 27). To our knowledge, quantitative assessment of drainage function at the level of the cervical lymph nodes in the setting of chronic hypertension and cerebral small vessel disease has not been fully investigated. Our hypothesis posited that SHRSP rats, with untreated chronic hypertension, would exhibit impaired dcLN drainage function as evidenced by impaired lymph flow.

For the cervical lymph node experiments, we used DCE-MRI with CSF administration of GadoSpin-P data acquired at the level of the rat’s neck (3, 28). Pathlines derived from the gSB analysis allowed for visualization of tracer-tagged lymph streams traversing the dcLNs. By integrating pathlines derived from gSB analysis with metrics such as speed, length, and tortuosity, we gained insight into the drainage function of the cervical lymph nodes in the context of chronic hypertension. The quantitative gSB analysis revealed that lymph tracer flow rates were reduced in the dcLNs of SHRSP rats. Intriguingly, we also noted that the pathlines, representing lymph streams, were longer and their course more complex and tortuous in the dcLNs of Wistar Kyoto (WKY) compared with SHRSP rats.

Overall, our study introduces a biologically informed gSB model and computational framework to investigate the drainage function of the cervical lymph nodes based on DCE-MRI images, offering the potential to better understand the directional relationships between systemic diseases, downstream drainage impairment, brain waste clearance, and ultimately neurodegeneration.

## Results

### Conventional analysis of the cervical lymph nodes reveals heterogeneous tracer uptake.

**Figure 1:**
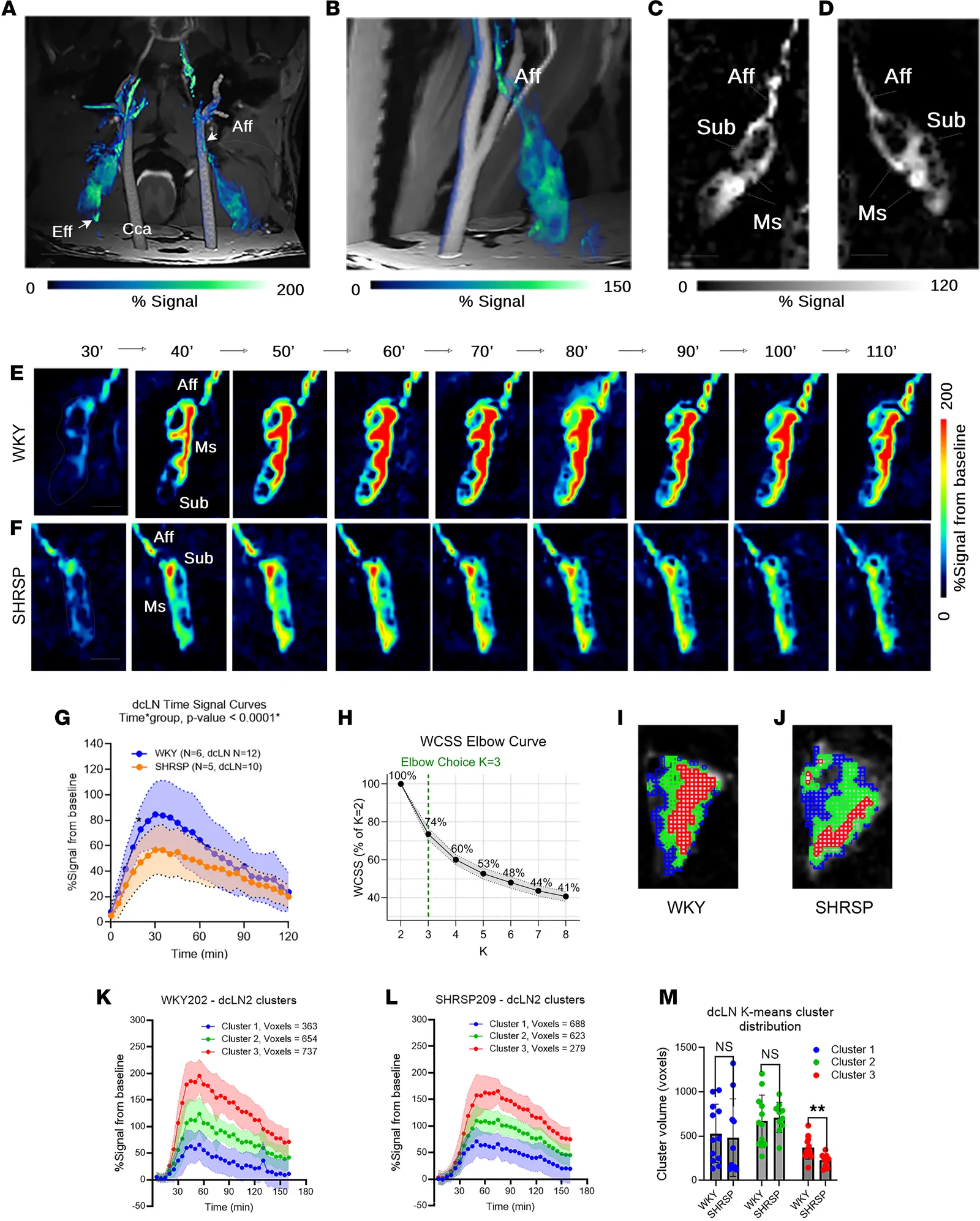
Reduced tracer transport in the dcLNs of hypertensive rats. (**A**) MIP of a dcLN from a WKY rat, with the location of the dcLN highlighted by a dashed circle. (**B**) Higher magnification of the dcLN. (**C** and **D**) Anatomical subcompartments, including the subcapsular (Sub) and medullary (Ms) sinuses. (**E** and **F**) Representative DCE-MRI time series from a WKY (**E**) and SHRSP (**F**) rat. (**G**) Mean TSCs for WKY (*n* = 6, 12 dcLNs) and SHRSP (*n* = 5, 10 dcLNs) rats, shown with 95% CI. (**H**) WCSS elbow curve for dcLN K-means cluster analysis. (**I** and **J**) Cluster analysis demonstrating the location of the subcapsular sinus (cluster 1, blue), paracortical sinus (cluster 2, green), and medullary sinus (cluster 3, red) in WKY and SHRSP dcLNs. (**K** and **L**) Cluster-specific TSCs from a WKY (**K**) and SHRSP (**L**) rat. (**M**) Volume fraction of the 3 clusters across both groups. Aff, afferent lymphatic vessel; Cca, common carotid artery; Eff, efferent lymphatic vessel. Scale bars: 2 mm (**A**–**F**), 1.5 mm (**I** and **J**). Data in **M** are presented as mean ± SD with individual data points shown. A linear mixed-effects model with FDR correction was used to assess group differences in TSCs (**G**), with the *P* value for time × group interaction indicated. A Mann-Whitney *U* test with FDR correction was used for the fractional volume analysis (**M**). ns, not significant; **P < 0.01.

To capture drainage of brain-derived fluids to the cervical lymph nodes, we implemented DCE-MRI with CSF administration of GadoSpin-P (MW 200 kDa) in WKY and SHRSP rats anesthetized with dexmedetomidine and low-dose isoflurane. A fraction of the GadoSpin-P–tagged CSF enters the glymphatic system and from there drains primarily to the dcLNs (3, 28). As such, the GadoSpin-P–tagged fluid from the brain arriving at the dcLN represents “lymph.” Therefore, by quantifying the tracer transport within the dcLN, we can estimate the lymph flow. To validate the metrics derived from the gSB model, we first conducted a conventional kinetic analysis of the DCE-MRI images to explore differences across WKY and SHRSP rats. Figure 1A shows a maximum intensity projection (MIP) image of the dcLN with interconnected signal streams overlaid on the corresponding anatomical MRI images from a normal rat. The dcLNs are positioned immediately lateral to the common carotid artery, typically at its bifurcation (Figure 1, A and B). The DCE-MRI images clearly showed the afferent and efferent lymphatic vessels in most dcLNs (Figure 1, A–D). The afferent signal streams were intermittently interrupted, reflecting the presence of valves in the lymphatic vessels. The stream associated with the afferent lymphatic vessel ran along the lingual artery branch from the external carotid artery in both WKY and SHRSP rats (Supplemental Figure 1, A and B; supplemental material available online with this article; https://doi.org/10.1172/jci.insight.206714DS1). The signal intensity distribution pattern in the dcLN revealed the location of the sinuses (Figure 1, C and D). It was not possible to clearly differentiate between the cortical and medullary sinuses. The medullary sinus was situated at the most medial aspect of the node and near the efferent lymphatic vessel. Additionally, circular low-signal-intensity regions were observed, representing the cell-rich follicles. These follicles are shielded units, wherein solutes larger than 70 kDa cannot penetrate (29, 30), preventing the entry of the larger 200 kDa GadoSpin-P tracer. In both normal WKY and SHRSP rats, 3D surface rendering of the anatomically segmented dcLNs showed that they were shaped like pointed triangular structures, with the base oriented toward the head and the apex pointing caudally (Supplemental Figure 1C). The afferent lymphatic vessels appeared to enter the dcLN at the base of the triangle; the efferent lymphatic vessel exited from the apex (Supplemental Figure 1C). The dcLNs in the SHRSP rats were more irregularly shaped, often with a wider triangular base. This shape allowed them to maintain their overall volume and surface area (Supplemental Figure 1, D and E) despite having a shorter length (Supplemental Figure 1F).

A dynamic DCE-MRI time series of dcLN images from representative WKY and SHRSP rats showed a signal increase approximately 30 minutes after CSF administration of GadoSpin-P, which signifies the arrival of brain-derived fluids (Figure 1, E and F). The average time for GadoSpin-P to arrive at the dcLN was similar across groups. The time was 28.3 ± 3.7 minutes for WKY (*n* = 7, dcLNs = 14) versus 29.5 ± 3.7 minutes for SHRSP (*n* = 5, dcLNs = 10) (mean difference = 1.3 minutes; 95% CI = –1.9, 4.5 minutes; *P* = 0.412). The time-signal curves (TSCs) for each dcLN were extracted, and the start time was defined as the moment when a significant increase in signal was detected. With this adjustment, the mean TSC for both groups was compared, demonstrating a significantly lower magnitude TSC in the SHRSP dcLN when compared with the WKY rats (Figure 1G, time × group interaction, *P* < 0.0001). We next asked whether the reduced dcLN drainage observed in the SHRSP rats was associated with other drainage abnormalities. For this purpose, the TSCs from the superficial cervical lymph nodes were extracted, and the analysis revealed no differences in tracer drainage across the 2 groups (Supplemental Figure 1, G and H).

To further quantify dcLN differences in tracer kinetic profiles across the 2 groups, we implemented a nonparametric *k*-means clustering approach. Prior to comparing cluster properties between groups, the optimal number of clusters *k* was determined empirically using within-cluster sum of squares (WCSS) elbow analysis applied to the voxel-wise time-activity curves of all WKY animals (see Methods for full details). The WCSS elbow curve demonstrated a substantial reduction in within-cluster variance through *k* = 3, which was therefore selected as the optimal number and applied uniformly to all subjects (Figure 1H). *k* = 3 clustering of the dcLN dynamic DCE-MRI data identified 3 kinetically distinct tissue compartments across all animals (Figure 1, I–L). Based on the temporal characteristics of the cluster time-activity curves, cluster 1 (blue), cluster 2 (green), and cluster 3 (red) exhibited progressively faster tracer uptake kinetics, corresponding spatially to the subcapsular sinus, paracortical sinus, and medullary sinus, respectively (Figure 1, I–L). Cluster 3 was specifically associated with the medullary sinus and hilum — the anatomical zone of efferent lymphatic output. The distribution of cluster voxel counts across groups is shown in Figure 1M. Total node volume did not differ between strains (WKY: 1568 ± 415 voxels; SHRSP: 1423 ± 494 voxels; Mann-Whitney *U* = 71, *P* = 0.489). Linear mixed-effects modeling (strain as fixed effect, animal as random intercept) revealed no significant difference between WKY and SHRSP in cluster 1 (mean difference = –47 voxels, *P* = 0.774) or cluster 2 (mean difference = 44 voxels, *P* = 0.750). In contrast, cluster 3 corresponding to the medullary sinus was significantly reduced in SHRSP compared with WKY (WKY: 368 ± 126 voxels; SHRSP: 226 ± 70 voxels; mean difference = −142 voxels, SE = 53, *P* = 0.008), suggestive of impaired efferent lymphatic drainage in hypertensive rats. Based on these data, we concluded that chronic hypertension is associated with reduced dcLN drainage function. However, conventional kinetic analysis does not allow quantification of lymph flow through the nodes, a limitation addressed by the gSB model below.

### The gSB model generates pathlines well-aligned with signal flows through the lymph nodes.

**Figure 2:**
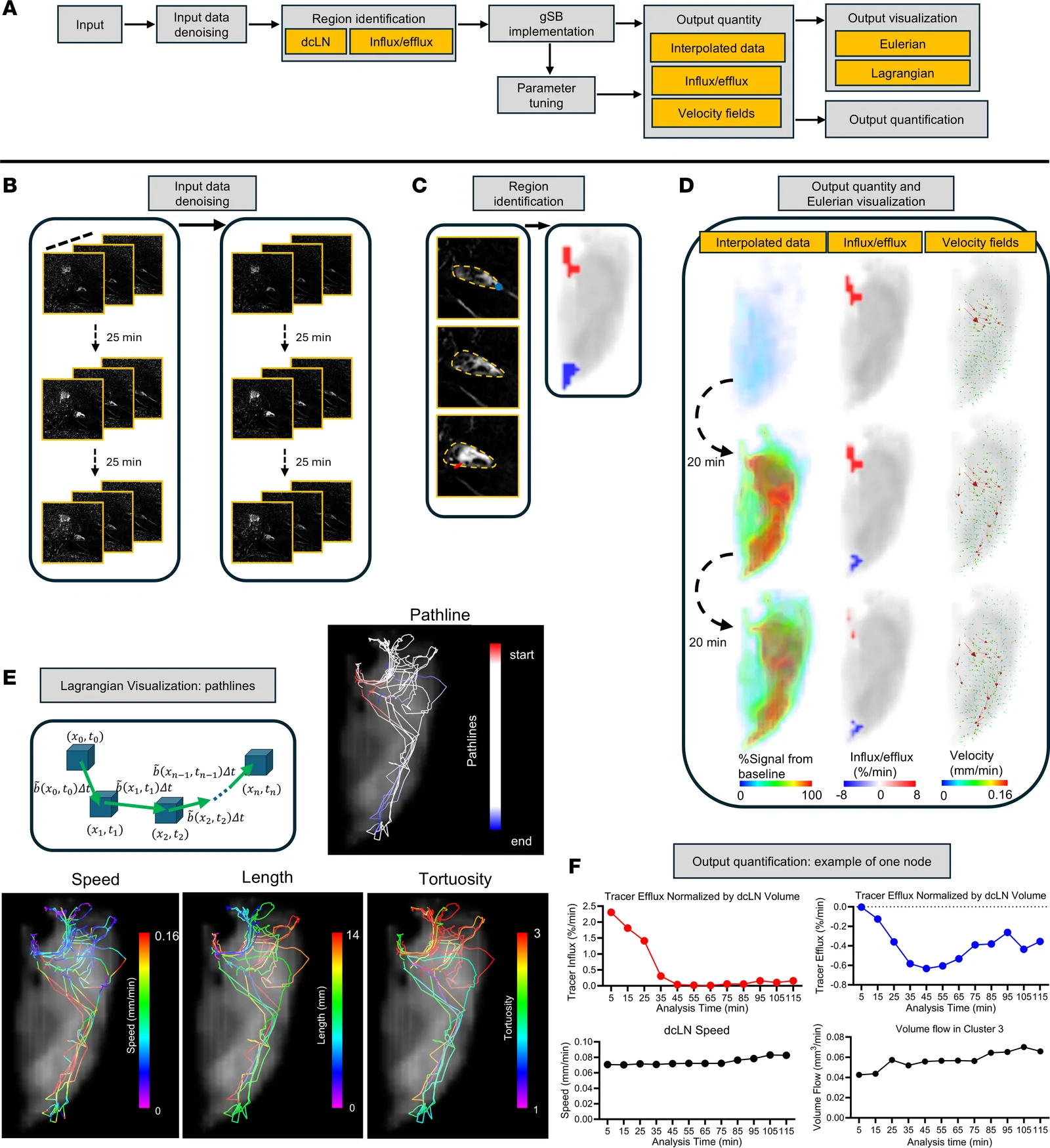
Computational pipeline for gSB analysis of dcLN DCE-MRI data. (**A**) Flowchart of the gSB model’s processing steps. (**B**) The input 3D MRI time series is denoised using a 4D affine diffusion filter. (**C**) Regions are identified, including the dcLN (orange), influx (red), and efflux (blue) areas. (**D**) Eulerian outputs from the model include interpolated images, influx/efflux maps, and velocity fields. (**E**) Lagrangian visualizations show tracer particle trajectories (pathlines), which can be endowed with metrics like speed, length, and tortuosity. (**F**) Key parameters quantified from the model include tracer influx, efflux, speed, and flow.

We designed a dedicated computational pipeline for the gSB analysis of lymph flow through the lymph node based on the DCE-MRI data (Figure 2, A–C). Throughout, “lymph flow” refers specifically to GadoSpin-P–tagged lymph transport as measured by DCE-MRI. The DCE-MRI images acquired with a spatial resolution of 0.008 mm^3^ and a 150 × 150 × 150 voxel matrix yielded a 3D dynamic time series of dcLN uptake and drainage of brain-derived fluids tagged with GadoSpin-P over approximately 2.5 hours (every 5 minutes, 32 frames in total). The analysis included several preprocessing steps, including denoising and segmentation of influx and efflux regions of the dcLN. The gSB model was applied on a rectangular cuboid that enclosed the dcLN region, and every second frame after the initial frame was fed into the algorithms with the analysis time window set to 120 minutes. The gSB model computed several output metrics: a time series of (a) interpolated data, (b) influx/efflux maps, and (c) velocity fields (Figure 2D and Supplemental Figure 2). Lagrangian visualizations were computed to track trajectories of individual particles — at the voxel level — based on density and velocity information. Each voxel within the dcLN gave birth to a pathline starting from that particular voxel. The pathlines were endowed with different metrics, including the flow property (speed) or the line property (length, tortuosity) (Figure 2E). Finally, in the Eulerian framework, quantification results yielded information of the gSB metrics at fixed locations in space over time and included time trajectories of (a) influx, (b) efflux, (c) speed, and (d) volume flow (Figure 2F).

### gSB analysis visualizes pathological dcLN microflows in hypertensive rats.

**Figure 3:**
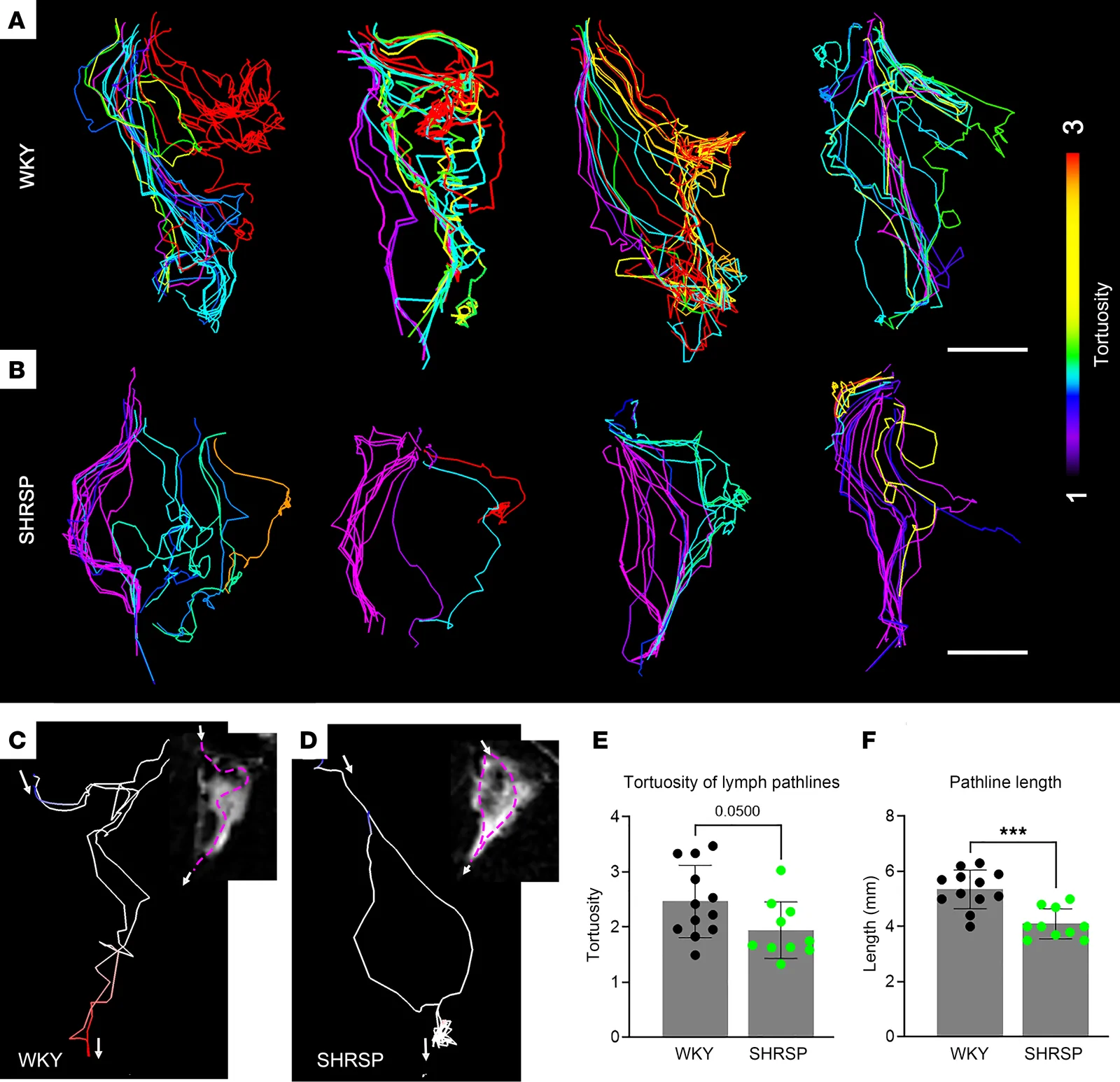
Tracer pathlines are more tortuous in the dcLNs of hypertensive rats. (**A** and **B**) Representative pathlines from 4 WKY (**A**) and 4 SHRSP (**B**) dcLNs, with pathline color indicating tortuosity. Scale bar: 2 mm. (**C** and **D**) Examples of highly tortuous pathlines from a single WKY (**C**) and SHRSP (**D**) dcLN. Insets show the pathlines overlaid on the corresponding DCE-MRI. (**E** and **F**) Quantification of pathline tortuosity (**E**) and length (**F**) for WKY (black) and SHRSP (green) rats. Data are presented as mean ± SD, with each dot representing a single dcLN. A 2-sided Welch’s *t* test was used for statistical comparison. *P* = 0.0500 (**E**); ***P < 0.0001 (**F**).

We first characterized the tortuosity and length of the pathlines traversing the dcLN across the 2 groups. The pathlines represent GadoSpin-P–tagged lymph streams at the voxel level captured over the 120-minute imaging period (Lagrangian framework). We selected 20 pathlines from each dcLN and color-coded them for tortuosity. In the WKY rats, the dcLN pathlines were longer and more tortuous compared with SHRSP rats (Figure 3). Pathlines from 4 dcLNs of WKY (Figure 3A) and SHRSP rats (Figure 3B) show more red, orange, and yellow pathlines, indicating increased tortuosity compared with SHRSP rats. The longest pathlines typically started in the uppermost zone of the subcapsular sinus, where the afferent lymphatic vessel merged with the dcLN and continued toward the efferent exit. The shorter pathlines started and ended in other parts of the cortical/medullary sinuses, and in WKY rats, they often appeared to exhibit a twirly course, circumnavigating the cell-rich follicles (Figure 3, A and C). In SHRSP rats, we observed that the pathlines were simpler and more linear (Figure 3, B and D). The quantitative analysis of the 20 pathlines selected revealed that the tortuosity of dcLN pathlines from WKY rats was increased by approximately 40% when compared with SHRSP rats. For WKY rats (*n* = 6, dcLNs = 12) tortuosity was 2.5 ± 0.2 versus 1.9 ± 0.2 for SHRSP rats (*n* = 5, dcLNs = 10) (mean difference = –0.5 ± 0.2; 95% CI = –1.0, 0.00008; *P* = 0.05). In accordance with these results, the overall dcLN pathline length was also documented to be significantly longer in WKY compared with SHRSP rats (*P* = 0.0001). From these data, we concluded that the intranodal course of lymph carrying the large MW GadoSpin-P tracer appears to be far more intricate and dynamic in normal rats compared with rats with chronic hypertension.

### gSB analysis reveals reduced dcLN lymph flow in hypertensive rats.

**Figure 4:**
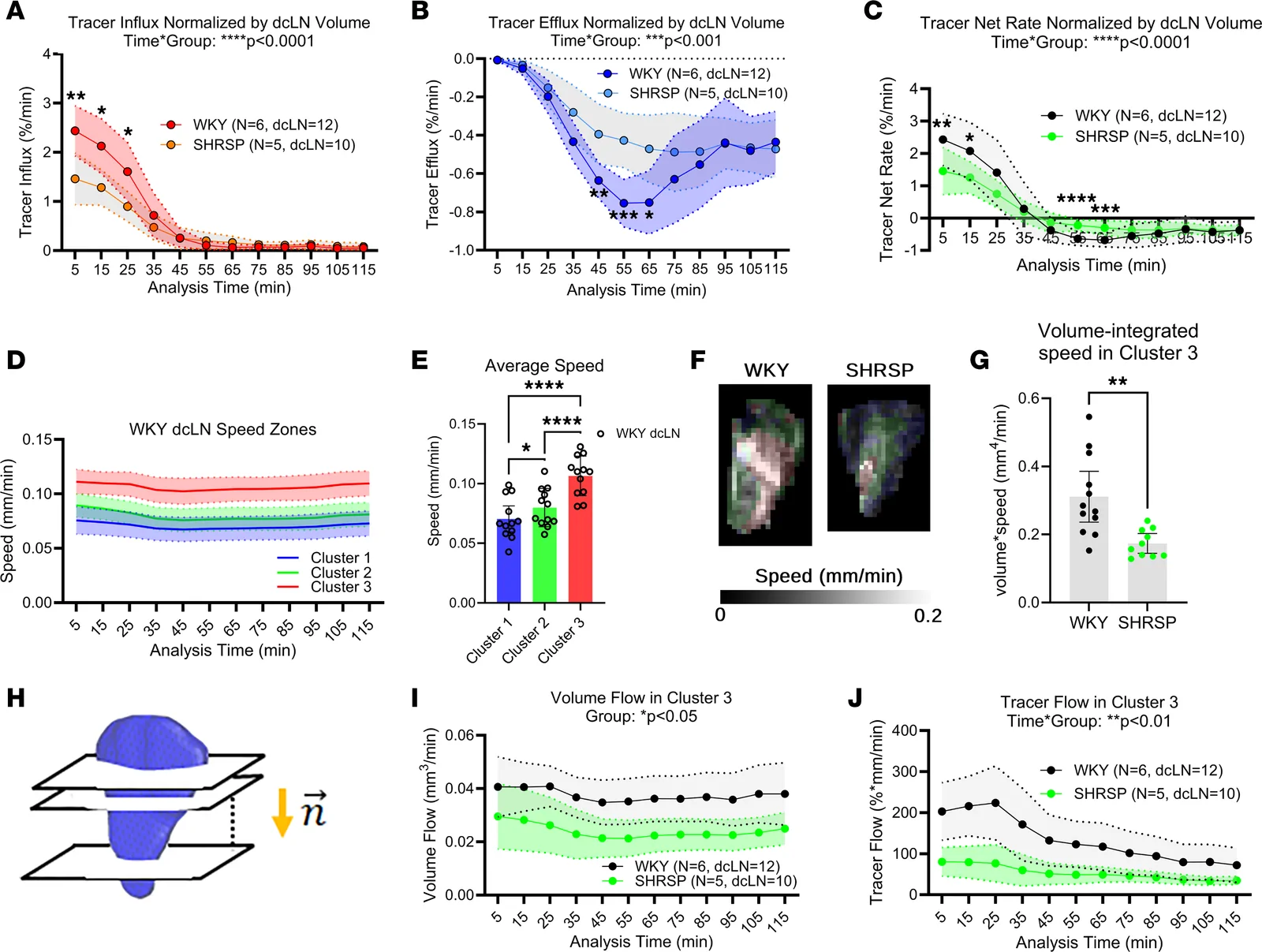
Lymph flow through the dcLNs is reduced in chronic hypertension. (**A**–**C**) Time course of volume-normalized tracer influx (**A**), efflux (**B**), and net rate (**C**) in dcLNs from WKY and SHRSP rats. (**D**) Time course of tracer speed within 3 *k*-means–derived clusters of the dcLN. (**E**) Comparison of average tracer speed across clusters for WKY dcLN. (**F**) Representative time-averaged speed map of a WKY dcLN and a SHRSP dcLN with clusters overlaid. (**G**) Comparison of volume-integrated speed (cluster volume × mean speed) in cluster 3 between WKY dcLNs and SHRSP dcLNs. (**H**) Schematic illustrating the calculation of tracer flow, where *n* is the vector perpendicular to the imaging slices. (**I**) Time course of volume flow through the cluster 3 region in WKY and SHRSP rats. (**J**) Time course of tracer flow through the cluster 3 region in WKY and SHRSP rats. Data in **A**–**C**, **I**, and **J** are presented as mean with 95% CI. Data in **D** are presented as mean with 95% CI for WKY dcLNs. Data in **E** and **G** are presented as mean ± SD with individual data points shown. In **A**–**C**, **I**, and **J**, group differences were assessed using a mixed-effects model with Geisser-Greenhouse correction, followed by Fisher’s least significant difference for multiple comparisons. In **E**, differences across clusters were assessed using unpaired *t* tests. In **G**, group differences were assessed using an unpaired *t* test. ns, not significant; *P < 0.05; **P < 0.01; ***P < 0.001; ****P < 0.0001.

To investigate the impact of chronic hypertension on lymph flow through the dcLN, we first derived and analyzed the time trajectories of tracer influx and efflux in the afferent and efferent regions, respectively, across the 2 groups. As anticipated, given that the lymph was tagged with GadoSpin-P administered as a bolus into the CSF, influx was initially high (Figure 4A), and over time when the tracer moved through the node and cleared, signal in the afferent region gradually dissipated (Figure 4A). Statistical analysis revealed that tracer influx was significantly reduced in SHRSP dcLNs compared with WKY rats (time × group interaction, *P* < 0.0001). These findings suggest that chronic hypertension impairs dcLN drainage through intrinsic structural or functional remodeling of the node, reduced upstream delivery of brain-derived lymph, or a combination of both. The efflux time trajectory of the normotensive WKY dcLN revealed that drainage was accelerated in the early period, peaked at approximately 55 minutes, and then gradually tapered off, although it was sustained throughout the remaining experimental time window (Figure 4B). For the SHRSP dcLN, the time course of efflux exhibited a steady acceleration, which plateaued at approximately 75 minutes. Statistical analysis showed that dcLN efflux was lower for SHRSP compared with WKY rats (time × group interaction, *P* < 0.001). We also calculated the net rate, which is the summation of tracer influx and efflux, providing information on the overall net flow through the nodes. These data revealed that the dcLN was dominated by tracer influx for the first 35 minutes, after which net efflux predominated (Figure 4C). The net rate time trajectories were significantly different across groups (time × group interaction, *P* < 0.0001).

We next quantified the time trajectories of gSB–derived speed within dcLN subregions defined by the *k*-means clusters. In WKY rats, speed was stable across the full experimental time window for all 3 clusters (Figure 4D), justifying the use of time-averaged speed as the primary metric. Comparison of mean cluster speeds in WKY rats revealed a spatial gradient of increasing transport velocity from the subcapsular sinus inward: cluster 1 (subcapsular sinus) exhibited the lowest average speed, approximately 12% lower than cluster 2 (paracortical sinus; *P* < 0.05); cluster 3 (medullary sinus) showed the highest speed, approximately 30% greater than cluster 2 (*P* < 0.0001; Figure 4E). Time-averaged speed maps overlaid with cluster masks confirmed that the high-speed region colocalized with cluster 3 in both WKY and SHRSP nodes (Figure 4F), consistent with the medullary sinus serving as the principal conduit for efferent lymphatic output. To assess dcLN functional capacity, we computed 3 complementary metrics within cluster 3. First, volume-integrated speed (cluster volume × mean speed; mm^4^/min) provided a scalar index of total fluid motion within the compartment and was significantly higher in WKY than SHRSP dcLNs (*P* < 0.01; Figure 4G). Because this metric does not directly correspond to lymph flow, we next computed directional volume flow through cluster 3 in the *xy* plane, averaged across slices spanning the central half of the node (from one-fourth to three-fourths of its height) to minimize edge effects (Figure 4H). Volume flow was stable over time in both groups and was significantly lower in SHRSP compared with WKY rats (group × time interaction, *P* < 0.05; Figure 4I), consistent with reduced lymphatic drainage capacity under chronic hypertension. Finally, given the spatial resolution of the DCE-MRI acquisition, individual voxels within cluster 3 may encompass both sinus lumen and immediately adjacent tissue, resulting in partial volume averaging. Tracer mass flow, which weights transport by local signal intensity, therefore, provides a more spatially sensitive index of lymph transport through the medullary sinus than volume flow alone. Tracer mass flow declined over time in both groups as the tracer cleared and was significantly lower in SHRSP than WKY dcLNs (group × time interaction, *P* < 0.01; Figure 4J), corroborating the volume flow findings and further supporting impaired efferent drainage in hypertensive rats.

### Histopathology.

**Figure 5:**
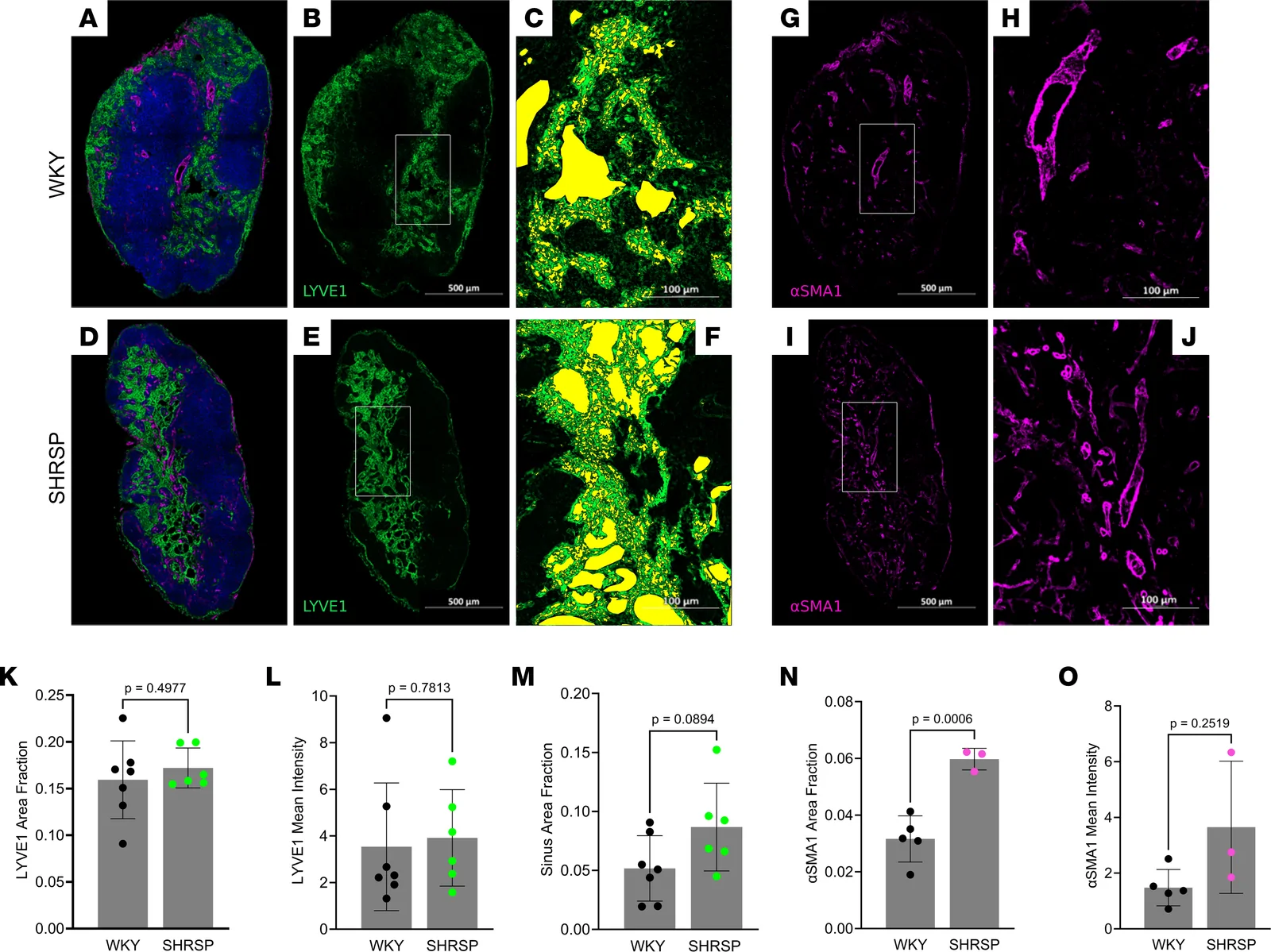
Histopathology of dcLNs from WKY and SHRSP rats. (**A**–**C**, **G**, and **H**) Representative WKY dcLN and (**D**–**F**, **I**, and **J**) SHRSP dcLN stained with LYVE-1 (green), α-SMA (magenta), and Hoechst (blue). The sinuses are color-coded in yellow in **C** and **F**. High-magnification insets of the boxed regions are shown in **C**, **F**, **H**, and **J**. (**K**–**O**) Quantification of LYVE-1 area fraction (**K**), LYVE-1 mean intensity (**L**), sinus area fraction (**M**), α-SMA area fraction (**N**), and α-SMA mean intensity (**O**) in dcLNs from WKY (black) and SHRSP rats (green in **K**–**M**; magenta in **N** and **O**). Each dot represents the value obtained from 1 dcLN. Data are presented as mean ± SD. Statistical analysis was performed using a 2-sided Welch’s unequal variances *t* test.

We performed IHC with structure-specific staining for the lymphatic sinuses (LYVE-1) and smooth muscle cell mass of the vasculature (α-smooth muscle actin, α-SMA) of dcLNs harvested from WKY and SHRSP rats. Figure 5 shows representative dcLNs stained for LYVE-1, highlighting the intricate network of lymphatic vessels traversing the lymph node in WKY (Figure 5, A and B) and SHRSP rats (Figure 5, D and E). We developed a comprehensive automated pipeline (SinusFinder) specifically designed for detecting and quantifying both the LYVE-1–labeled lymphatic surface (walls) and the inside sinus area (Supplemental Figure 3). An example of LYVE-1–positive sinus areas in WKY and SHRSP dcLNs is shown in the magnified figures (Figure 5, C and F). Figure 5 also shows the SMA distribution pattern in dcLNs of WKY and SHRSP rats (Figure 5, G–J). The quantitative analysis revealed that the LYVE-1–positive lymphatic surface area (normalized to the corresponding dcLN area) and the LYVE-1 mean intensity were similar across the WKY and SHRSP rats (Figure 5, K and L). However, the LYVE-1–positive sinus area was higher in the SHRSP compared with WKY rats (Figure 5M, *P* = 0.089), likely reflecting a larger number of ectasias observed in the dcLN of the hypertensive rats (Figure 5F). The quantitative analysis also revealed an increased area fraction and signal intensity of SMA associated with the dcLN arteries of the SHRSP rats, reflecting the chronic hypertensive conditions (Figure 5, N and O). Representative trichrome-stained sections from 1 WKY and 1 SHRSP dcLN are shown in Supplemental Figure 8, demonstrating striking fibrosis associated with the lymphatic sinuses in the SHRSP dcLN compared with the WKY control. Taken together, these findings indicate that chronic hypertension is associated with significant structural remodeling of the dcLN in SHRSP rats, including vascular changes consistent with those observed in other organs under hypertensive conditions.

## Discussion

Lymph nodes play a major role in regulating lymph transport, and excision of nodes can cause lymph edema (31, 32). In this work, we were interested in assessing dcLN function since these particular nodes serve as major hubs for receiving and draining the “dirty” fluid output from the brain (4, 5, 28, 33). A convergence of data indicates that the glymphatic and lymphatic systems are critically linked and work in concert to maintain cerebral homeostasis by facilitating CSF outflow and clearing metabolic waste from the brain (1, 14, 34, 35). Several studies have reported that CSF-glymphatic transport becomes disconnected from the lymphatic system in neurodegeneration (2, 28, 36, 37) and after mechanical impairment of cervical lymph node drainage (2, 3, 38). The clinical relevance of this connection is underscored in patients; individuals who undergo neck dissections of cervical lymph nodes for head and neck cancer treatment often present with facial lymphedema (39, 40) and can also develop cognitive impairment (41). Drainage from the brain to the cervical lymph nodes is generally assessed in a semi-quantitative manner by measuring the cumulative uptake of a given tracer after administration into the brain parenchyma or the CSF compartment. Although brain-derived tracer uptake into the cervical lymph nodes provides valuable information on glymphatic-lymphatic crosstalk, it does not directly measure lymph flow or dcLN drainage capacity. Existing mathematical models for estimating fluid flow in lymph nodes are physical models relying on simulation as well as information on hydraulic conductivity and pressure metrics, which are difficult to ascertain or nonexistent (42–45). To our knowledge, information on the functional drainage capacity of the cervical lymph nodes in the setting of systemic disease such as chronic hypertension has not been reported. In this work, we introduced a data-driven, biology-informed model, grounded in gSB theory, for estimating lymph fluid velocity in the dcLN from DCE-MRI data. Notably, this model operates on signal density information alone, bypassing the need for pressure and hydraulic metrics, and is applicable to both fluidics and porous media. Furthermore, the gSB model incorporates anatomical afferent and efferent lymphatic boundary information. For instance, we restricted the source to be positive at the afferent lymphatic vessel and negative at the efferent lymphatic vessel. The gSB model derived several biologically valuable metrics, including interpolated images, which allowed for visualization of real-time lymph trajectories through the dcLN.

The interpretation of GadoSpin-P tracer kinetics as a surrogate for lymphatic flow activity in the dcLN draws conceptual support from the established clinical paradigm of near-infrared fluorescence indocyanine green (NIRF-ICG) lymphangiography, in which the fluorescent signal of intradermally injected ICG transported through collecting lymphatics to draining nodes is interpreted as a measure of lymphatic transport capacity (46, 47). Quantitative NIRF-ICG parameters derived from this approach are used clinically to grade lymphatic dysfunction and monitor treatment response, validated in both human and preclinical models (46, 48). However, an important caveat applies to both techniques: neither ICG nor GadoSpin-P captures the full compositional complexity of lymph as characterized by its proteome and peptidome (49, 50). In the present study, the tracer kinetics therefore reflect the differential capacity of distinct compartments within the node — primarily the medullary sinuses — to take up, retain, and clear a macromolecular tracer, and should be interpreted as an index of functional drainage activity rather than a complete or direct characterization of lymph flow.

We used SHRSP rats to investigate the impact of chronic hypertension on the dcLN fluid flow. The *k*-means clustering analysis of the DCE-MRI data revealed that the WKY dcLN exhibited a larger cluster 3 compared with the SHRSP rats. The significant reduction in cluster 3 volume in SHRSP compared with WKY animals provides a structural and functional basis for impaired dcLN drainage in chronic hypertension. Cluster 3 is spatially localized to the medullary sinus, which converges at the hilum as the principal site of efferent lymphatic outflow. Given the large molecular weight of GadoSpin-P (200 kDa), the tracer is excluded from the parenchyma and confined to the lymphatic sinuses; the signal within cluster 3 therefore predominantly reflects sinus-resident tracer transport. A reduction in cluster 3 volume in SHRSP animals thus indicates structural compromise of the principal drainage pathway through the dcLN, consistent with the abnormal histopathological findings reported here.

Although the kinetic analysis was informative, quantitative metrics such as speed or drainage metrics could neither be derived nor visualized at the voxel level. In contrast, the gSB results provided visualization of lymph streams via pathlines and revealed abnormal drainage in the SHRSP rats in the form of reduced dcLN lymph flow. Compared with WKY rats, pathlines within the SHRSP dcLN were shorter and less tortuous, consistent with a more restricted transport network. The pathline analysis should be interpreted as exploratory and descriptive, providing visualization of transport patterns rather than definitive mechanistic evidence. Taken together, the convergent reductions in cluster 3 volume, volume flow, and tracer mass flow are consistent with impaired GadoSpin-P–tagged lymph transport through the medullary sinus in the hypertensive dcLN, suggesting reduced efferent drainage capacity along the tracer-accessible lymphatic pathway.

We acknowledge that reduced dcLN drainage in SHRSP could reflect upstream impairment of glymphatic transport, intrinsic nodal dysfunction, or both — and that these contributions cannot be fully disentangled with the current experimental design. Our previous work demonstrating reduced CSF and tissue solute speed in chronic hypertension (26) is consistent with a possible upstream contribution. In addition, the differential effect of the anesthesia on the SHRSP and WKY rats might have also contributed to the dcLN drainage differences. Nevertheless, the histopathological findings in the dcLN provide evidence that the node itself is structurally altered in SHRSP, supporting at least a partial intrinsic nodal component to the observed drainage impairment. Histopathology revealed more frequent sinus ectasias or dilated lymph sinuses, which has been associated with impaired lymph flow and lymph node atrophy (51). Although there is still a gap in our understanding of the underlying pathophysiology of dilated lymph sinuses, it is hypothesized that an increase in intralymphatic pressure, possibly due to downstream blockage of deeper lymphatic vessels, might play a role. We also documented increased collagen deposition at the level of the sinuses of the SHRSP dcLN. The increased α-SMA signal observed in SHRSP dcLNs likely reflects hypertension-driven vascular remodeling. In the SHRSP model, medial hypertrophy and arteriolosclerosis are well-established pathological features, consistent with the distribution of α-SMA staining observed here. Nevertheless, increased α-SMA signal could also reflect a greater number of vessels through angiogenesis. Because angiogenesis would be expected to involve increased microvessel density and endothelial proliferation, future studies incorporating endothelial markers will be required to distinguish vascular remodeling from angiogenesis. It is also important to acknowledge that the protein concentration of the lymph in afferent lymphatics across the WKY and SHRSP rats might have affected the differences in dcLN lymph flows. Specifically, early studies documented that the lymph node can alter lymph flow as well as lymph protein concentration under various conditions (52, 53). For example, lymph with low colloid osmotic pressure (low protein) becomes more concentrated as it passes through a node and loses fluid to the bloodstream. Currently, comprehensive data on plasma protein or colloid osmotic pressure within the lymph vasculature of SHRSP rats are not available in the literature, highlighting a clear need for future studies.

The temporal resolution of the DCE-MRI acquisition used here is coarser than label-free optical techniques, which offer subsecond to millisecond resolution, and clinical NIRF-ICG lymphangiography, which captures lymphatic transport in real time at 1–30 frames per second (54–57). However, optical methods are limited to superficial structures within approximately 1 cm of the tissue surface and cannot image the dcLNs without surgical exposure. MRI uniquely enables noninvasive imaging of deep structures, and although fast time-resolved angiography with interleaved stochastic trajectories (TWIST) sequences achieve 8- to 12-second temporal resolution for large vessels such as the thoracic duct (54, 58), the signal requirements for smaller structures necessitate longer acquisitions. The 5-minute frames used here are directly comparable to the sequences used in clinical DCE MR lymphangiography (0.5–5 minutes per acquisition) (54, 59) and represent a compromise between temporal resolution, imaging depth, and signal adequacy for dcLN characterization in the live rat.

We compared our lymph flow velocity results with values reported in the literature using other methods with higher temporal resolution. For example, Papadopoulos et al. (38) tracked fluorescently tagged nanoparticles transported from the CSF compartment toward the dcLN in mice and measured an oscillating velocity in the afferent vessels of approximately 6 mm/min. Separately, Blatter et al. (60) used B-mode Doppler optical coherence tomography imaging and reported a mean lymph flow velocity of approximately 3.5 mm/min in a mouse afferent vessel of the popliteal lymph node. Bouta et al. (61) used multiphoton fluorescence recovery after photobleaching to calculate lymph speed in the mouse afferent vessel of the popliteal lymph node, reporting a value of 6 mm/min. These reported lymph vessel velocities obtained in the mouse are several-fold higher than the velocities (i.e., 0.1 mm/min) we report within the rat lymph node using gSB analysis. This discrepancy is to be expected, as the transition of lymph from the narrow afferent vessel into the much larger cross-sectional area of the sinuses inevitably will reduce the bulk flow velocity. To overcome the discrepancy in the cross-sectional area, we compared the flows of the afferent lymphatic measured in previous studies with the flow dcLN values obtain in our study. For example, Blatter et al. reported an afferent flow value of 0.3 μL/h. We roughly estimated the flow through the rat dcLN by multiplying the speed value (approximately 0.1 mm/min) by the medullary sinus cross-sectional area (1 mm^2^), yielding a flow value of approximately 6 μL/h.

To our knowledge, the only class of models capable of deriving spatiotemporally resolved velocity fields and influx/efflux rates from DCE-MRI data is based on optimal mass transport (OMT) (8, 26, 62). The gSB framework is formally related to OMT but offers meaningful additional flexibility: it accommodates diffusion and allows for the random “creation” and “killing” (i.e., clearance or disappearance) of particles — features that are physically motivated in the context of lymphatic biology. It also permits the incorporation of prior information that can be calibrated against the data. These properties make gSB a more expressive framework than standard OMT for this application.

The modeling framework rests on 5 key assumptions: (a) image intensity is proportional to contrast solute concentration; (b) solute movement is governed by an advection-diffusion equation with a source term representing influx from afferent and efflux toward efferent lymphatic vessels; (c) the estimated concentration evolution minimizes relative entropy with respect to the prior evolution; (d) flow velocity is temporally stable, such that the velocity estimated at each time step serves as the prior for the subsequent step; and (e) the diffusion coefficient is constant, adopting the value from Chen et al. (28). The value and spatial distribution of the diffusion coefficient may influence the estimated velocity and influx/efflux results, representing a limitation of the current study. Future work should prioritize experimental or computational estimation of this parameter.

In this study, we focused on female rats for technical reasons: adult male WKY and SHRSP rats in the age range studied reach body weights of 400–500 g, which exceeds the practical limits of animal positioning and radiofrequency coil fitting within our 9.4T MRI system. We acknowledge that male SHRSP rats develop more severe hypertension than females (63), and on that basis we would expect hypertension-related pathology — including dcLN drainage impairment — to be at least as pronounced, and likely more severe, in males of the same age. The findings reported here may therefore represent a conservative estimate of the effect of chronic hypertension on dcLN lymphatic function. Future studies should include male animals to fully characterize the effect of sex on dcLN drainage in chronic hypertension.

In summary, we introduce gSB, a biology-informed computational fluid dynamics model, that quantifies lymph flow from DCE-MRI data by enforcing temporal stability. Applying gSB to hypertensive rats, we found a significant reduction in dcLN lymph flow, implicating chronic hypertension as a high-risk factor for impaired lymphatic drainage. This provides a quantitative platform to test the efficacy of antihypertensive therapies in restoring lymphatic function and for future refinement with complementary physiological data to improve the accuracy of the model.

## Methods

### Sex as a biological variable

Our study only examined female animals due to size restriction of the 9.4T MRI core used in the study, which cannot accommodate rats weighing more than 400 g.

### Rats

WKY (strain code 008) and SHRSP rats were purchased from Charles River Laboratories; the SHRSP line has since been discontinued and is maintained cryopreserved. The rats were housed in an environment with controlled temperature and humidity, individually ventilated cages, and a 12-hour light/12-hour dark cycle from 7:00 am to 7:00 pm, and they were fed standard chow and water ad libitum.

### gSB model

The SB models the most probable stochastic evolution of a closed system, such as a cloud of particles, from a known initial to a known final distribution over a specified period. The basic SB model considers that the total mass of the system remains unchanged. Building on Chen et al. (64, 65), who consider only the possibility of mass loss, we expanded the formulation of the model to account for microflow with both influx and efflux, generalizing the basic SB formalism into the gSB model. A detailed exposition building from SB to the gSB model is provided in the supplemental materials. Given an initial density function and a final density function, the gSB model arises as the solution to the following fluid flow optimization. Given an initial density function ρ_0_ and a final density function ρ_1_, the gSB model solves the following computational fluid dynamics problem:
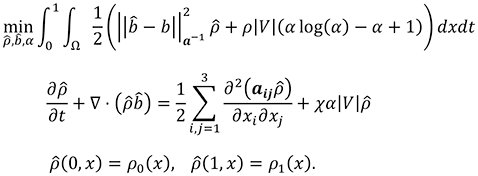


Here 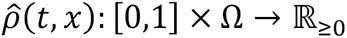

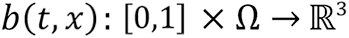

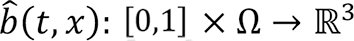
is the spatiotemporal dependent density function that can represent the concentration of the tracer; is the prior velocity field;

is the posterior velocity field;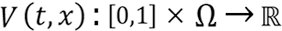 is the prior influx rate (*V* > 0) and efflux rate (*V* < 0);
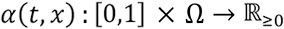

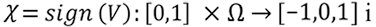
is the relative change in influx/efflux rate;is the indicator function of influx/efflux rate; Ω is the spatial domain, which is lymph node here;
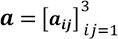

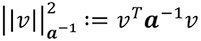
 is the diffusion coefficient matrix, and.

A point cloud illustration is shown in Supplemental Figure 4, A and B; observed initial and final particle distributions are shown in Supplemental Figure 4A, and Supplemental Figure 4B shows gSB-generated particle trajectories and intermediate distributions. The gSB model provides the most likely distribution of flow velocities as well as influx and efflux rates. The prior modelencodes any hypotheses on the flow of solute in, across, and out of the flow field, and serves as a starting point to obtain a posterior model that aligns more closely with any available dataset that is being observed. An illustration that juxtaposes the schematics of the distribution of prior and posterior flow lines is in Supplemental Figure 4, C and D. To highlight the effect of the choice of different priors, a 1D simulation and its parameters are provided in Supplemental Figure 5 (Supplemental Videos 1–3) and Supplemental Table 1. The parameters for implementing gSB on lymph node data are provided in Supplemental Table 2.

### Animals and DCE-MRI imaging

#### Anesthesia and CSF catheter.

On the day of the experiment, the rats underwent anesthesia, and a small catheter was placed into the CSF compartment via the cisterna magna (28). Anesthesia included induction with 3% isoflurane delivered into a chamber in 100% oxygen. After the loss of the righting reflex, the rats were given dexmedetomidine (0.007 mg/kg i.p.) mixed with glycopyrrolate (0.02 mg/kg i.p.). During surgery, isoflurane was kept at 2% delivered with a 1:1 mixture of O_2_/air. In a stereotaxic instrument and with the head flexed 30°, a small catheter made of copper (0.32 mm o.d., Nippon Tokushukan) connected to PE10 tubing was carefully inserted into the cisterna magnum and secured in place using cyanoacrylate glue. After surgery, the anesthetized rats were transferred to the MRI instrument and placed supine on the custom-built 3D-printed animal bed. During imaging, anesthesia was maintained with a subcutaneous infusion of dexmedetomidine (0.009 mg/kg/h) supplemented with low-dose 1% isoflurane. Throughout the experiments, vital signs including pulse oximetry, heart rate, respiratory rate, and body temperature were monitored using noninvasive MRI-compatible monitors (SA Instruments).

#### DCE-MRI imaging.

All MRI imaging was performed on a Bruker 9.4T/16 magnet (Bruker BioSpin) operating with Paravision 6.0.1 software and interfaced with an Avance III-HD console. We used a custom-made transmit radio-frequency coil (50 mm i.d.) for signal excitation, and a 2 cm surface radio-frequency loop coil (Bruker) positioned over the neck of the rat was used as a receiver. After baseline scanning, 30 μL of GadoSpin-P was infused into CSF at the rate of 1.5 μL/min using a high-precision pump (Legato 130 Nanoliter, KD Scientific). The lyophilized powder of GadoSpin-P was reconstituted in 850 μL of sterile saline (0.9% NaCl), yielding a 25 mM GadoSpin-P solution and used within 12 hours of preparation. For DCE-MRI, T1-weighted images were acquired using a flow-compensated 3D FastLow-Angle-Shot (FLASH) sequence (TR/TE/FA/number of averages = 15 ms/4 ms/15°/1, scanning time = 5.63 minutes, resolution = 0.2 × 0.2 × 0.2 mm^3^). Postcontrast 3D FLASH T1-weighted scans were acquired continuously for 180 minutes. For each rat, the DCE-MRI time series included 37 images with a size of 150 × 150 × 150.

### Preprocessing of DCE-MRI images.

The DCE-MRI data were corrected for motion, followed by intensity normalization and smoothing, and then voxel-wise percentage signal change to baseline was calculated using the submandibular jaw muscles for signal intensity normalization. Next, the DCE-MRI data underwent further denoising. For the denoising step, the series of MRI images was treated as a 4D image with a matrix of 150 × 150 × 150 × 37, and a 4D affine denoising filter to smooth the spatial and temporal domains was applied simultaneously, using the 2D version (66) and 3D version (67). The level set surface evolved along the affine-invariant geometric flow:
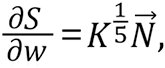


where *S* = (*x*(*w*), *y*(*w*), *z*(*w*), *t*(*w*)) was the level set surface, *w* was the pseudo time parameter in the surface evolution, *K* was the Gaussian curvature, and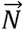 as the unit normal vector.

The corresponding change in image intensity would be:


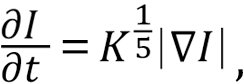


where *I* = *I*(*x*, *y*, *z*, *t*, *w*) was the image intensity at location (*x, y, z*), time *t*, and evolution step *w*. In the implementation, we used the finite difference method to solve the above partial differential equation. The space resolution was Δ*x* = Δ*y* = Δ*z* = Δ*t* = 1. The length of the evolution step was Δ*w* = 0.1, and 10 iterations were run. The denoised percentage signal change from baseline images was then used for the remaining analysis.

### Anatomical dcLN segmentation.

The first step in processing dynamic DCE-MRI images involves reviewing the cine sequence. This step allowed us to inspect for any leakage of GadoSpin-P contrast from the CSF compartment, as well as to identify severe artifacts or irregularities. Ensuring these issues were addressed helped ensure that the subsequent gSB analysis was conducted on data with a reliable contrast enhancement pattern. This step allowed us to include 12 dcLNs in the WKY rats (*n* = 1 rat, dcLN = 2 was excluded due to excessive motion) and 10 dcLN in the SHRSP rats (*n* = 1 rat, dcLN = 2 were excluded due to failure of the GadoSpin-P injection). Second, the time series of postcontrast T1-weighted images were summed and used as an anatomical template to manually outline volumes of interest using PMOD software (v3.908, PMOD Technologies). We then manually identified and segmented the dcLN, the influx afferent region, and the efflux efferent region on the time-summed image. By checking the signal curve for each node, we selected a time window that started at the time the tracer arrived in the dcLN and included 120 minutes of the experimental time window. The DCE-MRI data within this timeframe were ingested into the gSB algorithm, and we refer to this time period as analysis time. In addition, we also segmented the accessory cervical lymph nodes and extracted the TSCs from all the rats.

### Clustering.

We performed *k*-means clustering on the denoised DCE-MRI data in MATLAB to delineate functional zones within each dcLN. For each node, voxels in the lowest 20% by signal “energy” (defined as the sum of squared signal intensity over time) were designated as background and excluded from clustering. The number of clusters was selected using the elbow method applied to the WCSS applied on all WKY dcLNs (68). Cluster indices were sorted according to the mean signal intensity of each cluster to ensure consistency across all nodes. Specifically, cluster 1 was assigned to the lowest-signal region, whereas cluster 3 was assigned to the highest-signal region.

## Lagrangian visualization.

We followed Koundal et al. (26) to derive the Lagrangian visualization. Each pathline in Figure 2E is a function of time and starting point, and we denote this function by *L*(*t,* ***x***), which satisfies
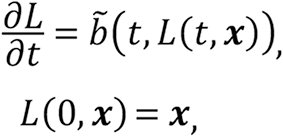


where 
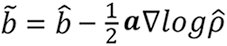
is the augmented velocity derived from the gSB results. In our study, we assume *a* is a constant matrix. The augmented velocity comes from writing the advection-diffusion equation in conservation form:
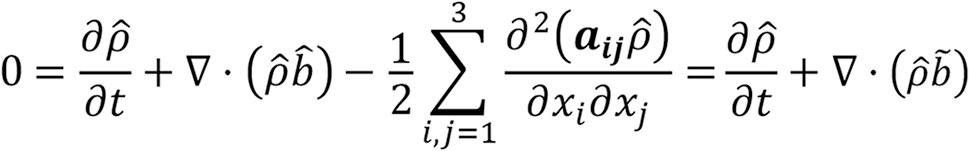


Once the pathlines were derived, we endowed them with different properties, such as length *l*(*L*), tortuosity *τ*(*L*), or other flow quantities for its corresponding time *t* and location *L*(*t,* ***x***), like speed. The tortuosity was derived from dividing the length by the distance between the starting point and endpoint, i.e., *τ*(*L*) = *l*(*L*)/|*L*(end time, *x*) – *x*|

The pathlines in Figure 2E and Figure 3, A, B, E, and F, were selected according to their initial points within the influx mask. Specifically, the 3D influx mask was first reshaped into a 1D array, and 20 starting voxels were then selected at approximately equal intervals from this array. In cases where the influx mask contained fewer than 20 voxels, all available voxels were used.

## Histopathology

A separate series of WKY (*n* = 7) and SHRSP (*n* = 6) was used for IHC of the dcLNs. The rats were anesthetized with an overdose of ketamine and xylazine and perfusion-fixed with 4% paraformaldehyde. The harvested dcLNs were subjected to increasing concentrations (10%, 20%, 30%) of sucrose in PBS and cryopreserved in OCT compound (Sakura Finetek). Next, 40 μm thick sections were cut using a Leica CM1950 cryostat. These sections were permeabilized using 0.25% Triton X-100 containing PBS for 5 minutes and blocked in 1% blocking buffer for 30 minutes. Immunofluorescence staining was performed on these sections to detect LYVE-1 and α-SMA1 expression. Although all nodes were processed with LYVE-1, SMA staining was carried out on a subset of the available samples. For this, cryosections of dcLNs were incubated overnight with primary antibodies: sheep LYVE-1 (1:100, R&D Systems, AF7939) and mouse α-SMA (1:100, Invitrogen, MA5-11547). After primary antibody incubation, these sections were washed 3 times with PBS, PBS-Tween, and PBS. Further, these sections were incubated for 1 hour and 30 minutes with respective secondary antibodies: Alexa Fluor 488–conjugated donkey anti-sheep IgG (1:300, Invitrogen, A11015), and Alexa Fluor 647–conjugated donkey anti-mouse IgG (1:300, Invitrogen, A32787). After secondary incubation, sections underwent 3 additional washes with PBS, PBS-Tween, and PBS. Tissue sections were mounted on glass slides using a drop of CitiFluor MWL 4-88 (Electron Microscopy Sciences, 17977-150) antifade reagent. Images of stained sections were acquired at 200× original magnification using a fluorescence microscope (Zeiss, Axio Imager Z2).

Paraffin-embedded dcLN sections were stained with Masson’s trichrome following standard protocols to assess collagen deposition and tissue fibrosis. Sections were imaged using a Zeiss Axio Imager Z2 bright-field microscope.

## Histopathology image segmentation and quantification

The pipeline of LYVE-1 image processing (SinusFinder) included 4 major steps: (a) preprocessing, where LYVE-1 images are standardized, patched, and binarized; (b) deep learning branch, which applies a U-Net model (69) for semantic prediction of sinus structures; (c) connectivity branch, which employs binarization and connected component analysis (70) (57) to recover cavities that may be missed by the deep learning branch; (d) fusion and quantitative analysis, where results are merged, filtered by tissue masks, and converted into interpretable morphological quantities. An overview of this pipeline is shown in Supplemental Figure 3, the key parameter used is provided in Supplemental Table 3, and a detailed explanation is included in the supplemental materials. We also performed post hoc processing by segmenting large sinus areas that the above analysis did not pick up manually.

The segmentation of the whole node was done manually, from which we could derive the area of that node slice. The LYVE-1/α-SMA1 area considers the voxels with LYVE-1/α-SMA1 intensity 25 inside the node. The LYVE-1/α-SMA1 mean intensity is the averaged intensity value over the node.

## Quantification of influx and efflux

We denote the number of voxels within the dcLN as *nVoxels*. We denote the influx region and the clearance region by Ω_+_ and Ω_−_. The tracer influx, normalized by the dcLN volume, is expressed as
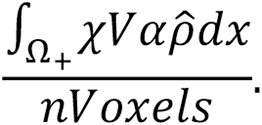


Similarly, the tracer efflux, normalized by dcLN volume, and the tracer net rate are:


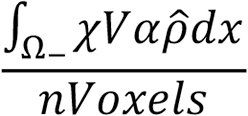


and


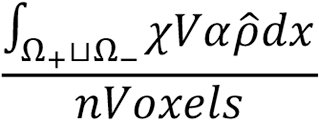


respectively. The speed value is an averaged value weighted by the density, that is,


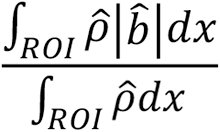


Considering the number of particles that each pathline represents, the pathline tortuosity (length) value is computed by averaging the tortuosity (length) value of each pathline weighted by the signal at its starting point, where the signal is the mean of the first 2 frames.

The tracer flow is computed at each slice parallel to the *xy-*plane and is averaged over the slices from the top to the bottom. Specifically, if we assume the *z* index of the top point of the dcLN is *z_0_* and the *z* index of the bottom point of the dcLN is *z*_1_, then the starting slice has *z* index


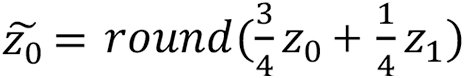


and the bottom slice has *z* index


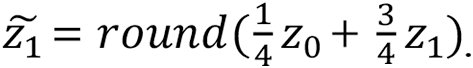


The tracer flow is computed by


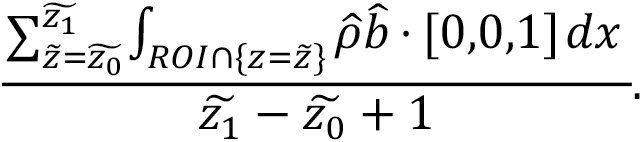


Online supplemental material

The Supplemental methods include sections for (a) the SB model, (b) the gSB variant and biologically informed gSB model implementation, and (c) the LYVE-1 segmentation methodology. Supplemental Figure 1 shows the drainage pathways and MRI morphology of the cervical lymph nodes. Supplemental Figure 2 shows the visual display of the gSB model output derived from the lymph nodes. Supplemental Figure 3 shows the computational pipeline for LYVE-1 imaging processing. Supplemental Figure 4 shows a conceptual illustration of pathlines derived from the gSB model highlighting the importance of prior information. Supplemental Figure 5 shows 1D simulations directly illustrating the importance of prior information. Supplemental Figure 6 shows a comparison between skipped frames and gSB interpolations. Supplemental Figure 7 shows sensitivity analysis results. Supplemental Figure 8 shows trichrome stains of dcLNs from a WKY and SHRSP rat.

## Statistics

Data are presented as mean and 95% CI. For each time trajectory curve, a 3-point equal-weight moving average was performed. For each of the gSB time trajectories of tracer influx, efflux, net rate, speed or flow, as well as the TSC derived from the DCE-MRI images, a linear mixed-effects model with a heterogeneous variance-covariance matrix for repeated measures with independent variables including group (WKY vs. SHRSP), time, and the time × group interaction, and animal as a random effect was fit to compare the mean differences of different outcomes between groups and between time points after GadoSpin-P appearance in dcLNs. Group differences were calculated using a post hoc pairwise Fisher’s least significant difference test.

Two-group comparisons between groups (WKY vs. SHRSP) were made using a 2-tailed, unpaired, independent *t* test assuming unequal variances. Voxel counts per cluster were analyzed for each dcLN individually. The rationale for this analysis is based on evidence that individual lymph nodes within the same anatomical drainage region may differ functionally (71). To account for the nonindependence of paired nodes within the same subject, a linear mixed-effects model was fitted with strain as a fixed effect and animal as a random effect, where each animal contributed 2 dcLN observations. All statistical analyses were performed using IBM SPSS Statistics Premium 31 and GraphPad Prism 10.4.2. Graph plotting was performed using GraphPad Prism 10.4.2, VisIt 3.4.1, MATLAB, and ImageJ (NIH). A *P* value of less than 0.05 was chosen to indicate statistical significance. All statistics are reported in the figure legends.

## Study approval

All the animal work was approved by the IACUC at Yale University, New Haven, Connecticut, USA.

## Data availability

All data supporting the findings of this study are available within the article and its supplemental materials, including the Supporting Data Values file containing all underlying data values for figures and statistical analyses. The original DCE-MRI data and associated metadata are publicly available in Zenodo at https://zenodo.org/records/22232880 The gSB computational code and cluster analysis code are publicly available on GitHub at https://github.com/KaimingXu98/uSB_CFD_demo (branch: main; commit: ec7e6f934c314484ae7a91163777546c528f910b; https://github.com/KaimingXu98/uSB_CFD_demo/commit/ec7e6f934c314484ae7a91163777546c528f910b).

## Author contributions

KX with assistance from TTG designed the computational algorithms for the SB model, performed the gSB analysis of the data, and wrote the gSB Supplemental methods section. SK performed all the DCE-MRI experiments on the rats. DCE-MRI data analysis was performed by HB. AB performed the histopathology on the lymph nodes. QR and CY performed the quantitative analysis using SinusFinder on the lymph nodes. XP provided intellectual contribution and helped interpret the data. KX, HB, and TTG conceived the study, oversaw data analysis, and wrote the manuscript. The statistical analysis was performed by HB, KX, and AB.

## Conflict of interest

The authors have declared that no conflict of interest exists.

## Funding support

This work is the result of NIH funding, in whole or in part, and is subject to the NIH Public Access Policy. Through acceptance of this federal funding, the NIH has been given a right to make the work publicly available in PubMed Central.

- NIH/National Heart, Lung, and Blood Institute grant 1R01HL176017-01 (to HB).
- Leducq Foundation (23CVD03).

## Supplementary Material

Supplemental data

Supporting data values

## Version 1 08/12/2026

In-Press Preview

## Version 2 09/22/2026

Electronic publication
