# Supplementary material for: Chronic hypertension impairs lymphatic drainage in deep cervical lymph nodes
Source: JCI Insight. 2026 Aug 11;11(18):e206714. doi: 10.1172/jci.insight.206714 (PMC13596731; doi:10.1172/jci.insight.206714)
Supplement: Supplementary file 1 [file jciinsight-11-206714-s340.pdf]

Supplemental Material

Chronic Hypertension Impairs Lymphatic Drainage in Deep Cervical Lymph Nodes

Kaiming Xu, Ankita Bhardwaj, Sunil Koundal, Qin Ren, Chenyu You, Xenophon Papademetris, Helene Benveniste, Tryphon T. Georgiou

Contents

1 Supplemental Method **3**

1.1 Schrödinger's Bridge model 3

1.2 Biologically-informed generalized SB model 4

1.3 Biologically-informed gSB model implementation 6

1.4 Comparison between interpolation and skipped data 11

1.5 Sensitivity analysis 11

1.6 LYVE-1 segmentation methodology 11

2 Supplemental Figures **14**

3 Supplemental Tables **23**

3.1 Table S1: Parameters table of 1D simulation 23

3.2 Table S2: Parameters table of Lymph node study 23

3.3 Table S3: Summary of LYVE-1 Segmentation Parameters 23

1 Supplemental Method

1.1 Schrödinger's Bridge model

The Schrödinger Bridge (SB) models the most probable stochastic evolution of a closed system, such as a cloud of particles, from a known initial to a known final distribution over a specified period. The basis of Schrödinger's model is a diffusion process:

$$dX_t = b(t, X_t)dt + \sigma(t, X_t)dW_t \tag{Eqn-S1}$$

in Euclidean space, where $X_t \in \mathbb{R}^3$ represents the location of particles that drift along under the influence of forces modeled by a drift term $b(t, X_t) \in \mathbb{R}^3$ and stochastic excitation with noise (diffusion) coefficient $\sigma(t, X_t) \in \mathbb{R}^{3\times3}$. The stochastic excitation is modeled as a standard Wiener process – W_t has increments dW_t that are independent and normally distributed with zero mean and variance dt. The probability law of X_t is encoded in $b(t, \cdot)$ and $\sigma(t, \cdot)$, and constitutes the *prior* probability distribution; it is to be understood as a rule for assigning probability to paths that particles can take.

Schrödinger's thought-experiment conceived of a large number of particles diffusing according to equation (Eqn-S1), with their distribution being recorded at two points in time, $t = 0$ and $t = 1$. The respective probability density functions at these two points in time, denoted by $\rho_0(x)$ and $\rho_1(x)$, are referred to as time-marginals, since the time at which they describe the spread of the ensemble of particles is specified. It is standard that the time-marginals $\rho(t, x)$ of the process X_t in equation (Eqn-S1) obey the so-called Fokker-Planck partial differential equation:

$$\frac{\partial \rho(t, x)}{\partial t} = -\nabla \cdot (b(t, x)\rho(t, x)) + \frac{1}{2}\sum_{i,j=1}^{3} \frac{\partial^2(\mathbf{a}_{ij}\rho(t, x))}{\partial x_i \partial x_j}, \tag{Eqn-S2}$$

where $\nabla\cdot$ denotes the divergence operator, and where the matrix $[\mathbf{a}_{ij}]_{ij=1}^3 := \sigma(t, x)\sigma(t, x)^\top$ is assumed to be a positive definite, with $^\top$ denoting the transpose. Knowing the starting distribution $\rho(0, x) = \rho_0(x)$, the terminal distribution $\rho(1, x)$ is completely specified by equation (Eqn-S2). But what if it happens that data on the position of particles at the terminal time, giving $\rho_1(x)$ as the empirical distribution of particles, conflicts with the model in that:

$$\rho(1, x) \neq \rho_1(x)? \tag{Eqn-S3}$$

The essence of Schrödinger's question is that $\rho_1(x)$ is only an *empirical* marginal, i.e., the distribution of a finite-collection of particles. The central limit theorem does not apply, and hence, there is no reason why equation (Eqn-S3) must hold with equality. The inherent randomness has conspired to drive the particles along a different route than what the *prior* law encoded in equation (Eqn-S2) prescribed. Thence, Schrödinger asked for *the most likely path* that the particles took so as to reconcile the experimental data in $\rho_0(x)$ and $\rho_1(x)$.

The likelihood of particles traversing paths that are not favored by the nominal model from equation (Eqn-S1) (equivalently, by the prior probability law), can be expressed in terms of the relative entropy between the posterior and the prior law – this is known as Sanov's theorem (1). Thus, the SB problem amounts to finding a posterior probability law $\widehat{\mathcal{P}}$ so that the relative entropy between posterior and prior $\mathcal{P}$, formally:

$$H(\widehat{\mathcal{P}}|\mathcal{P}) := \int_{\text{paths}} \log\left(\frac{d\widehat{\mathcal{P}}}{d\mathcal{P}}\right) d\widehat{\mathcal{P}}(\omega),$$

is minimal; abstractly, the probability laws are to be understood as probability density functions on the space of paths $\omega = \{X_t \mid t \in [0,1]\}$ – a space that contains all continuous functions. This integral over a large space where 'points' are complete paths, can be re-written in a computationally accessible form of an (action) integral in time and space, directly in terms of the drift $b(t,x)$ in the dynamics, described by equation (Eqn-S1), of the prior, and the sought drift $\widehat{b}(t,x)$ and spatio-temporal probability density $\widehat{\rho}(t,x)$ of the posterior, namely[1],

$$H(\widehat{\mathcal{P}}|\mathcal{P}) = \frac{1}{2}\int_0^1 \int_{\mathbb{R}^3} \|\widehat{b}(t,x) - b(t,x)\|^2_{\mathbf{a}^{-1}} \widehat{\rho}(t,x) dx dt,$$

where $a = \sigma\sigma^\top$ as before, $\|v\|^2_{\mathbf{a}^{-1}} := v^\top \mathbf{a}^{-1} v$, and $\widehat{b}, \widehat{\rho}$ obey together a Fokker-Planck equation as in equation (Eqn-S2).

As a result, the 'most likely' probability law $\widehat{\mathcal{P}}$, in the sense of being the closest to the prior and restores consistency with experimental data, that may include initial and terminal marginal distributions that conflict with the prior, can be obtained by minimizing $H(\widehat{\mathcal{P}}|\mathcal{P})$ with respect to $\widehat{b}$ under the constraint that it matches the experimental data. The revised drift term $\widehat{b}(t,x)$ defines the solution to Schrödinger's problem. The problem can now be formulated as the following constrained optimization problem:

$$\min_{\widehat{\rho},\widehat{b}} \frac{1}{2}\int_0^1 \int_{\mathbb{R}^3} \|\widehat{b} - b\|^2_{\mathbf{a}^{-1}} \widehat{\rho} dx dt, \tag{Eqn-S4a}$$

$$\frac{\partial \widehat{\rho}}{\partial t} + \nabla \cdot (\widehat{\rho}\widehat{b}) = \frac{1}{2}\sum_{i,j=1}^{3} \frac{\partial^2(\mathbf{a}_{ij}\widehat{\rho})}{\partial x_i \partial x_j}, \tag{Eqn-S4b}$$

$$\widehat{\rho}(0,x) = \rho_0(x), \quad \widehat{\rho}(1,x) = \rho_1(x). \tag{Eqn-S4c}$$

It is a rather fortuitous fact that a change of parameters from density and 'velocity' $(\widehat{\rho}, \widehat{b})$ to density and 'momentum' $(\widehat{\rho}, \widehat{m} := \widehat{b} \times \widehat{\rho})$, makes the optimization problem convex, opening up a variety of ways to efficiently solve the problem, see e.g., (1, 2).

1.2 Biologically-informed generalized SB model

The physiology of transportation within biological tissue, as in tracing a plume of solute in the lymph, departs from Schrödinger's basic paradigm in a variety of ways, most importantly, in that a solute enters and exits gradually, and in that the experimentally estimated distributions at the two end-points rarely match. The basic SB model can thereby be enhanced to reflect the underlying physiology. We use the term generalized SB (gSB) to highlight the fact that the terminal distributions are no longer constrained to have equal mass, since mass flows in and out over suitable subdomains, and that small amounts may be partly even unaccounted for.

From a mathematical standpoint, source and sink pools where solute particles originate and dissipate, can be modeled as discrete sites, or even as a single site, allowing particles to flow in and out of the domain. Physiology on the other hand allows us to further delineate spatial subdomains where solute flows in (afferent) and flows out (efferent). We introduce a stochastic model for the underlying mechanism, as in SB, where initial

[1] Here, we assume that the marginal distribution $\rho(0,x)$ at the starting point in time is the same for the prior and posterior probability law.

(prior) estimates for respective rates can be adjusted to the most likely values (posterior) that account for the available experimental data. To this end, we consolidate the prior rates into a single function $V(t,x)$, taking positive sign in the afferent region and negative in the efferent. In this way, equation (Eqn-S4b) in the optimization problem described in equation (Eqn-S4) is replaced with:

$$\frac{\partial \widehat{\rho}}{\partial t} + \nabla \cdot (\widehat{\rho}\, \widehat{b}) = \frac{1}{2} \sum_{i,j=1}^{3} \frac{\partial^2 (\mathbf{a}_{ij} \widehat{\rho})}{\partial x_i \partial x_j} + \widehat{V} \widehat{\rho}. \qquad \text{(Eqn-S4b}'\text{)}$$

The gSB model was discussed in Chen *el al.*(3, 4), though only for lossy domains ($V < 0$).

The posterior probability law is once again sought to minimize the likelihood function $H(\widehat{\mathcal{P}}|\mathcal{P})$, that now includes an added term for the mechanism of inflow and outflow. This involves the prior and posterior rates, V and $\widehat{V}$, respectively. We assume that the afferent and efferent regions are fixed as specified in the nominal model, and thus, equivalently, that the ratio $\alpha := \widehat{V}/V$ is positive for all (t,x). The likelihood term is

$$h(\alpha) := \int_0^1 \int_{\mathbb{R}^3} \widehat{\rho} |V| (\alpha \log(\alpha) - \alpha + 1)) dx dt,$$

comparing prior to posterior rates across the domain. In total, the gSB variant of Schrödinger's stochastic model, that accounts for influx and efflux at regions specified by the sign of V, is cast as the optimization problem

$$\min_{\widehat{\rho}, \widehat{b}, \alpha} \int_0^1 \int_{\mathbb{R}^3} \frac{1}{2} \left(\|\widehat{b} - b\|^2_{\mathbf{a}^{-1}} \widehat{\rho} \right) dx dt + h(\alpha) \qquad \text{(Eqn-S4a}'\text{)}$$

subject to equation (Eqn-S4b′) and the boundary conditions from equation (Eqn-S4c) imposed by the experimental data, i.e., as (Eqn-S4a′-Eqn-S4b′-Eqn-S4c). The optimization takes place over $\widehat{\rho}(t,x)$, $\widehat{b}(t,x)$ and $\alpha(t,x) \geq 0$.

Next, we turn to biologically-informed gSB models in three dimensions (3D) over *regions of interest* (ROI), which are typically denoted by the symbol Ω, such that $\Omega \subset \mathbb{R}^3$. We divide the ROI into three subregions, $\Omega = \Omega_+ \sqcup \Omega_- \sqcup \Omega_0$; Ω_+ is the afferent region where influx takes place, Ω_- is the efflux region where the solute exits the ROI, and lastly, Ω_0 is a region where the solute only advects and diffuses. We assume that no chemical reaction takes place within Ω that may alter the nature and mass of the solute, and thereby, that any variability in the mass of the solute over the window of time when observations are made is due to influx and efflux in the respective regions.

We proceed with a re-parametrization of the gSB model and of the corresponding optimization problem, with variable substitutions to more clearly reflect physical quantities; the reformulation seeks to simplify computational tasks. We first define the indicator function

$$\chi(x) := \mathrm{sign}(V(0,x)) = \begin{cases} 1 & x \in \Omega_+ \\ -1 & x \in \Omega_- \\ 0 & x \in \Omega_0 \end{cases} \qquad \text{(Eqn-S5)}$$

and incorporate this into the optimization problem (equations Eqn-S4a′-Eqn-S4b′-Eqn-S4c), namely, we have:

gSB-optimization:

$$\min_{\widehat{\rho},\widehat{b},\alpha} \int_0^1 \int_{\mathbb{R}^3} \frac{1}{2}\left(\|\widehat{b}-b\|^2_{a^{-1}}\widehat{\rho}\right) dxdt + h(\alpha)$$

$$\frac{\partial\widehat{\rho}}{\partial t} + \nabla\cdot(\widehat{\rho}\,\widehat{b}) = \frac{1}{2}\sum_{i,j=1}^{3}\frac{\partial^2(\mathbf{a}_{ij}\widehat{\rho})}{\partial x_i\partial x_j} + \chi\alpha|V|\widehat{\rho}$$

$$\widehat{\rho}(0,x) = \rho_0(x), \quad \widehat{\rho}(1,x) = \rho_1(x).$$

where $\widehat{\rho} : [0,1]\times\mathbb{R}^3 \to \mathbb{R}_{\geq 0}$ is the density function, $\widehat{b} : [0,1]\times\mathbb{R}^3 \to \mathbb{R}^3$ is the velocity vector field, $\alpha : [0,1]\times\mathbb{R}^3 \to \mathbb{R}_{>0}$ is change of the influx and efflux rate, $b : [0,1]\times\mathbb{R}^3 \to \mathbb{R}^3$ is the 'prior' velocity vector field, $V : [0,1]\times\mathbb{R}^3 \to \mathbb{R}$ is the prior rates of the influx and efflux, $a = [a_{ij}]^3_{ij=1}$ is a positive definite diffusion coefficient matrix, $\chi : \mathbb{R}^3 \to \{-1,0,1\}$ is the indicator function used to specify the influx/efflux region,

$$h(\alpha) := \int_0^1 \int_{\mathbb{R}^3} \widehat{\rho}|V|(\alpha\log(\alpha) - \alpha + 1))dxdt,$$

$$\|v\|^2_{a^{-1}} := v^\top a^{-1} v.$$

1.3 Biologically-informed gSB model implementation

Change of Variables

To avoid $\alpha|V|$ from being negative in the numerical process, we replaced it by an exponential function $e^{\widehat{R}}$ and performed optimization on a new variable $\widehat{R}$. We also assume the diffusion process is isotropic so that we set $a = 2DI$ where I is a 3×3 identity matrix.

The revised formulation is as follows:

$$\begin{aligned}
&\min_{\widehat{\rho},\widehat{b},\widehat{R}} \int_0^1 \int_{\mathbb{R}^3} \frac{1}{4D}\left(\|\widehat{b}-b\|^2\widehat{\rho}\right) dxdt + h(e^{\widehat{R}}/|V|)\\
&\frac{\partial\widehat{\rho}}{\partial t} + \nabla\cdot(\widehat{\rho}\widehat{b}) = D\Delta\widehat{\rho} + \chi e^{\widehat{R}}\widehat{\rho}\\
&\widehat{\rho}(0,x) = \rho_0(x), \quad \widehat{\rho}(1,x) = \rho_1(x),
\end{aligned} \tag{Eqn-S6}$$

Model implementation

In this section, we explain how we implement gSB model in detail. Given a series of images $I_0, I_1, \ldots, I_n$, we solve the following fluid dynamics reformation of the Schrödinger Bridge for each pair of consecutive images I_j and I_{j+1}:

$$\begin{aligned}
&\inf_{\widehat{\rho}^j,\widehat{b}^j,\widehat{R}^j} \int_0^T \int_\Omega \frac{1}{4D}\widehat{\rho}^j|\widehat{b}^j - b^j|^2 dxdt + \beta\int_\Omega(\widehat{\rho}^j(T,x) - I_{j+1})^2 dx\\
&\quad + \int_0^T\int_\Omega \widehat{\rho}^j(\widehat{R}^j e^{\widehat{R}^j} - e^{\widehat{R}^j} - e^{\widehat{R}^j}\log|V^j| + |V^j|)dxdt,\\
&\frac{\partial\widehat{\rho}^j}{\partial t} + \nabla\cdot(\widehat{\rho}^j\widehat{b}^j) = D\Delta\widehat{\rho}^j + \chi\widehat{\rho}^j e^{\widehat{R}^j},\\
&\widehat{\rho}^j(0,x) = \tilde{I}_j(x).
\end{aligned} \tag{Eqn-S7}$$

Here $\widehat{\rho}^j : [0,T]\times\Omega \to \mathbb{R}_{\geq 0}$ is the family of mass density defining the interpolations from I_j to I_{j+1}, $\widehat{b} : [0,T]\times\Omega \to \mathbb{R}^3$ is the velocity fields from I_j to I_{j+1}, $e^{R_j} : [0,T]\times\Omega \to \mathbb{R}_{>0}$

is the magnitude of the influx and efflux rate from I_j to I_{j+1}. The initial marginal is either the first image or the final time interpolation from the last step, i.e. $\tilde{I}_0(\cdot) = I_0(\cdot)$ and $\tilde{I}_j(\cdot) = \widehat{\rho}^{j-1}(T, \cdot), j = 1, 2, \ldots, n-1$. The prior of the velocity is either 0 or the velocity computed from the previous step, i.e. $b^0 = 0$ and $b^j = \widehat{b}^{j-1}, j = 1, 2, \ldots, n-1$. The reason we set the prior of velocity like above is that the bulk flow has the property that it does not change much in time. The prior of the source magnitude is either set as a constant V_0 or the source computed from the previous step, i.e. $V^0 = V_0$ and $V^j = e^{\widehat{R}^{j-1}}, j = 1, 2, \ldots, n-1$. We also wanted to apply the source information from the previous step to the current step.

Compared to formulation (Eqn-S6), we specify the spatial domain as Ω, generalize the temporal domain to $[0, T]$, and apply the fact $V = \chi|V|$. We also remove the final marginal condition and add an error term in the objective function since we do not want to match the noise in the images. For the analysis region Ω, we use a rectangular cuboid enclosing the ROI. The distance between each side and the ROI is 10 voxels. The density outside of the ROI is 0. We implement open boundary conditions, which allow nonzero normal velocity at the boundary, considering the difficulty of implementing a strict close boundary condition. A large analysis region and the error term in the objective function make it almost impossible for the mass to move out of the analysis region.

We use Gauss-Newton methods to solve the optimization problem (Eqn-S7). The algorithm is as follows (Algorithm 1). Here we denote the objective function by $\Gamma^j(\widehat{\rho}^j, \widehat{b}^j, \widehat{R}^j)$. Specifically,

$$\begin{aligned}\Gamma^j(\widehat{\rho}^j, \widehat{b}^j, \widehat{R}^j) = &\int_0^T \int_\Omega \frac{1}{4D} \widehat{\rho}^j |\widehat{b}^j - b^j|^2 dx dt + \beta \int_\Omega (\widehat{\rho}^j(T, x) - I_{j+1})^2 dx \\ &+ \int_0^T \int_\Omega \widehat{\rho}^j (\widehat{R}^j e^{\widehat{R}^j} - e^{\widehat{R}^j} - e^{\widehat{R}^j} \log |V^j| + |V^j|) dx dt\end{aligned}$$

Algorithm 1 gSB algorithm

Load $\tilde{I}_j, I_{j+1}, V_0, \chi$, and other parameters;
Prior: if $j = 0$, then $[|V^0|, b^0] = [V_0, 0]$; if $j > 0$, then $[|V^j|, b^j] = [\exp(\widehat{R}^{j-1}), \widehat{b}^{j-1}]$;
Initial guess: $[\widehat{R}^j, \widehat{b}^j] = [log(|V^j|), b^j]$;
for $i = 1, 2, \ldots,$maxIteration **do**
  Compute interpolations $\widehat{\rho}^j$ from the advection-diffusion-source equation: $\widehat{\rho}^j = advDiffSrc(\tilde{I}_j, \widehat{b}^j, \widehat{R}^j, \chi)$;
  Compute the gradient g and Hessian H of Γ^j with respect to $[\widehat{b}^j; \widehat{R}^j]$;
  Solve linear system $Hs = -g$ for descent step s;
  Do line search for step size l.
  if line search fails **then**
    return $[\widehat{b}^j; \widehat{R}^j]$;
  end if$[\widehat{b}^j; \widehat{R}^j] = [\widehat{b}^j; \widehat{R}^j] + ls$;
end for
return $[\widehat{b}^j; \widehat{R}^j]$;

Numerical Methods

Discretization We only explain the discretization on 3D domain here. The 1D case is a simple generalization. Since the image is already a good discretization of the spatial domain, we followed the image voxels and divide the analysis rectangle cuboid domain Ω into a cell-centered grid with size $n_x \times n_y \times n_z$ and uniform spacing $\Delta x, \Delta y$, and Δz. Let $n = n_x n_y n_z$ be the total number of cells (voxels). Time is divided into m intervals of length Δt with $m+1$ time steps. Moreover, the superscript 0 corresponds to initial time $t = 0$, m corresponds to final time $t = 1$ and $\Delta t \times m = T$. We use $\widehat{\boldsymbol{\rho}}^{\boldsymbol{j}} = [\widehat{\boldsymbol{\rho}}_{\boldsymbol{0}}^{\boldsymbol{j}}; \ldots; \widehat{\boldsymbol{\rho}}_{\boldsymbol{m}}^{\boldsymbol{j}}]$, $\widehat{\boldsymbol{b}}^{\boldsymbol{j}} = [\widehat{\boldsymbol{b}}_{\boldsymbol{1}}^{\boldsymbol{j}}; \ldots; \widehat{\boldsymbol{b}}_{\boldsymbol{m}}^{\boldsymbol{j}}]^T$, $\widehat{\boldsymbol{R}}^{\boldsymbol{j}} = [\widehat{\boldsymbol{R}}_{\boldsymbol{1}}^{\boldsymbol{j}}; \ldots; \widehat{\boldsymbol{R}}_{\boldsymbol{m}}^{\boldsymbol{j}}]^T$ to represent temporal-spatial discretized $\widehat{\rho}^j$, $\widehat{b}^j$, and $\widehat{R}^j$, respectively. These bold letters are all column vectors. The length of $\widehat{\boldsymbol{\rho}}_{\boldsymbol{i}}^{\boldsymbol{j}}, \widehat{\boldsymbol{R}}_{\boldsymbol{i}}^{\boldsymbol{j}}$ are all n, and $\widehat{\boldsymbol{b}}_{\boldsymbol{i}}^{\boldsymbol{j}}$ has a length of $3n$. Note that the velocity $\widehat{\boldsymbol{b}}_{\boldsymbol{i}}^{\boldsymbol{j}}$ describes the velocity field from $(i-1)^{\text{th}}$ time step to i^{th} time step. For convenience, we remove the superscript $\boldsymbol{j}$ for $\widehat{\boldsymbol{\rho}}^{\boldsymbol{j}}, \widehat{\boldsymbol{b}}^{\boldsymbol{j}}$, and $\widehat{\boldsymbol{R}}^{\boldsymbol{j}}$ in the following context and use $\boldsymbol{I}$ to represent discretized version of final time marginal I_{j+1}.

*Objective function The objective function is discretized in

$$\begin{aligned}\Gamma_s(\widehat{\boldsymbol{\rho}}, \widehat{\boldsymbol{b}}, \widehat{\boldsymbol{R}}) &= \frac{1}{4D}(\Delta x \Delta y \Delta z \Delta t)[\widehat{\boldsymbol{\rho}}^T M((\widehat{\boldsymbol{b}} - \boldsymbol{b}) \odot (\widehat{\boldsymbol{b}} - \boldsymbol{b})) \\ &+ \widehat{\boldsymbol{\rho}}^T(\widehat{\boldsymbol{R}} \odot \exp(\widehat{\boldsymbol{R}}) - \exp(\widehat{\boldsymbol{R}}) - \exp(\widehat{\boldsymbol{R}})\log(|\boldsymbol{V}|) + |\boldsymbol{V}|)] \\ &+ \beta(\Delta x \Delta y \Delta z)\|\widehat{\boldsymbol{\rho}}_{\boldsymbol{m}} - \boldsymbol{I}\|^2,\end{aligned} \tag{Eqn-S8}$$

where $M = (I_m \otimes [I_n|I_n|I_n])$, $\otimes$ is the Kronecker product, and $\odot$ is the Hadamard product. The objective function Γ_s is a function of only $\widehat{\boldsymbol{b}}$ and $\widehat{\boldsymbol{R}}$ since $\widehat{\boldsymbol{\rho}}$ can be derived from $\widehat{\boldsymbol{b}}$ and $\widehat{\boldsymbol{R}}$ via the advection diffusion source equation.

Advection-diffusion-source equation The advection-diffusion-source equation is discretized and solved in the following three steps:

$$\frac{1}{\Delta t}(\widehat{\boldsymbol{\rho}}_{\boldsymbol{i}}^{\boldsymbol{src}} - \widehat{\boldsymbol{\rho}}_{\boldsymbol{i}}) = \boldsymbol{\chi}_{\boldsymbol{i}} \odot \widehat{\boldsymbol{\rho}}_{\boldsymbol{i}} \odot \exp \widehat{\boldsymbol{R}}_{\boldsymbol{i}}, \tag{Eqn-S9}$$

$$\widehat{\boldsymbol{\rho}}_{\boldsymbol{i}}^{\boldsymbol{adv}} = S(\widehat{\boldsymbol{b}}_{\boldsymbol{i}})\widehat{\boldsymbol{\rho}}_{\boldsymbol{i}}^{\boldsymbol{src}}, \tag{Eqn-S10}$$

$$\frac{1}{\Delta t}(\widehat{\boldsymbol{\rho}}_{\boldsymbol{i+1}} - \widehat{\boldsymbol{\rho}}_{\boldsymbol{i}}^{\boldsymbol{adv}}) = DQ\widehat{\boldsymbol{\rho}}_{\boldsymbol{i+1}}. \tag{Eqn-S11}$$

Here exp is an element-wisely exponential operator, $S(\widehat{\boldsymbol{b}}_{\boldsymbol{i}})$ is the averaging matrix linear to $\widehat{\boldsymbol{b}}_{\boldsymbol{i}}$ using the particle-in-cell method (5) which interpolated the transported mass by its nearest neighbors, Q is the discretization of the diffusion operator Δ, and $\odot$ is the Hadamard product.

Combining all three equations above, we have

$$\widehat{\boldsymbol{\rho}}_{\boldsymbol{i+1}} = L^{-1}S(\widehat{\boldsymbol{b}}_{\boldsymbol{i}})T(\widehat{\boldsymbol{R}}_{\boldsymbol{i}}, \boldsymbol{\chi}_{\boldsymbol{i}})\widehat{\boldsymbol{\rho}}_{\boldsymbol{i}}, \tag{Eqn-S12}$$

where $L = I_n - DQ\Delta t$, and $T(\widehat{\boldsymbol{R}}_{\boldsymbol{i}}, \boldsymbol{\chi}_{\boldsymbol{i}}) = I_n + \text{diag}(\boldsymbol{\chi}_{\boldsymbol{i}} \odot \exp \widehat{\boldsymbol{R}}_{\boldsymbol{i}})\Delta t$. Then, we recursively solve for $\widehat{\boldsymbol{\rho}}_{\boldsymbol{i+1}}$
using $\widehat{\boldsymbol{\rho}}_{\boldsymbol{0}}, (\widehat{\boldsymbol{b}}_{\boldsymbol{1}}, \widehat{\boldsymbol{R}}_{\boldsymbol{1}}), (\widehat{\boldsymbol{b}}_{\boldsymbol{2}}, \widehat{\boldsymbol{R}}_{\boldsymbol{2}}), \ldots, (\widehat{\boldsymbol{b}}_{\boldsymbol{i}}, \widehat{\boldsymbol{R}}_{\boldsymbol{i}})$.

For simplicity, we denote $A(\widehat{\boldsymbol{b}}_{\boldsymbol{i}}, \widehat{\boldsymbol{R}}_{\boldsymbol{i}}, \boldsymbol{\chi}_{\boldsymbol{i}}) = L^{-1}S(\widehat{\boldsymbol{b}}_{\boldsymbol{i}})T(\widehat{\boldsymbol{R}}_{\boldsymbol{i}}, \boldsymbol{\chi}_{\boldsymbol{i}})$ and equation (Eqn-S12) becomes

$$\widehat{\boldsymbol{\rho}}_{\boldsymbol{i+1}} = A(\widehat{\boldsymbol{b}}_{\boldsymbol{i}}, \widehat{\boldsymbol{R}}_{\boldsymbol{i}}, \boldsymbol{\chi}_{\boldsymbol{i}})\widehat{\boldsymbol{\rho}}_{\boldsymbol{i}}. \tag{Eqn-S13}$$

Gradient and Hessian To apply the Gauss-Newton minimization procedure, we need expressions for the gradient $g(\widehat{b}, \widehat{R})$ and the Hessian $H(\widehat{b}, \widehat{R})$. Before that, we first calculate the derivative of $\widehat{\boldsymbol{\rho}}$ with respect to $(\widehat{\boldsymbol{b}}, \widehat{\boldsymbol{R}})$ and we call it J. The matrix J is formulated as follows:

$$J = (J_{\widehat{\boldsymbol{b}}}, J_{\widehat{\boldsymbol{R}}}) = ((J^{k,j}_{\widehat{\boldsymbol{b}}}), (J^{k,j}_{\widehat{\boldsymbol{R}}})) = ((\frac{\partial \widehat{\boldsymbol{\rho}}_{\boldsymbol{k}}}{\partial \widehat{\boldsymbol{b}}_{\boldsymbol{j}}}), (\frac{\partial \widehat{\boldsymbol{\rho}}_{\boldsymbol{k}}}{\partial \widehat{\boldsymbol{R}}_{\boldsymbol{j}}})). \quad \text{(Eqn-S14)}$$

Recursively, we can extend equation (Eqn-S13) as

$$\widehat{\boldsymbol{\rho}}_{\boldsymbol{k}} = A(\widehat{\boldsymbol{b}}_{\boldsymbol{k-1}}, \widehat{\boldsymbol{R}}_{\boldsymbol{k-1}}, \boldsymbol{\chi}_{\boldsymbol{k-1}}) \dots A(\widehat{\boldsymbol{b}}_{\boldsymbol{j}}, \widehat{\boldsymbol{R}}_{\boldsymbol{j}}, \boldsymbol{\chi}_{\boldsymbol{j}}) \widehat{\boldsymbol{\rho}}_{\boldsymbol{j}}. \quad \text{(Eqn-S15)}$$

Then we can calculate the derivatives in equation (Eqn-S14)

$$\frac{\partial \widehat{\boldsymbol{\rho}}_{\boldsymbol{k}}}{\partial \widehat{\boldsymbol{b}}_{\boldsymbol{j}}} = \begin{cases} A(\widehat{\boldsymbol{b}}_{\boldsymbol{k-1}}, \widehat{\boldsymbol{R}}_{\boldsymbol{k-1}}, \boldsymbol{\chi}_{\boldsymbol{k-1}}) \dots & \\ \dots L^{-1} \frac{dS(\widehat{\boldsymbol{b}}_j)}{d\widehat{\boldsymbol{b}}_{\boldsymbol{j}}} T(\widehat{\boldsymbol{R}}_{\boldsymbol{j}}, \boldsymbol{\chi}_{\boldsymbol{j}})_{\boldsymbol{j}} & k > j \\ 0 & k \leq j, \end{cases} \quad \text{(Eqn-S16)}$$

$$\frac{\partial \widehat{\boldsymbol{\rho}}_{\boldsymbol{k}}}{\partial \widehat{\boldsymbol{b}}_{\boldsymbol{j}}} = \begin{cases} L^{-1} S(\widehat{\boldsymbol{b}}_{\boldsymbol{k-1}}) T(\widehat{\boldsymbol{R}}_{\boldsymbol{k-1}}, \boldsymbol{\chi}_{\boldsymbol{k-1}}) \dots & \\ \dots L^{-1} S(\widehat{\boldsymbol{b}}_{\boldsymbol{j}}) \Delta t \,\mathrm{diag}(\boldsymbol{\chi}_{\boldsymbol{i}} \odot \exp \widehat{\boldsymbol{R}})_{\boldsymbol{j}} & k > j \\ 0 & k \leq j. \end{cases} \quad \text{(Eqn-S17)}$$

Now we focus on calculating the gradient $g(\widehat{\boldsymbol{b}}, \widehat{\boldsymbol{R}})$:

$$g = [g_{\widehat{\boldsymbol{b}}}; g_{\widehat{\boldsymbol{R}}}] = [\frac{\partial \Gamma}{\partial \widehat{\boldsymbol{b}}}; \frac{\partial \Gamma}{\partial \widehat{\boldsymbol{R}}}]. \quad \text{(Eqn-S18)}$$

Taking the gradient of Γ with respect to $\widehat{\boldsymbol{b}}$ and $\widehat{\boldsymbol{R}}$, we have

$$\begin{aligned} g_{\widehat{\boldsymbol{b}}} = \frac{\partial \Gamma_s}{\partial \widehat{\boldsymbol{b}}} &= (\Delta x \Delta y \Delta z \Delta t)(2(M \mathrm{diag}(\widehat{\boldsymbol{b}} - \boldsymbol{b})^T \widehat{\boldsymbol{\rho}}) + J^T_{\widehat{\boldsymbol{b}}} M((\widehat{\boldsymbol{b}} - \boldsymbol{b}) \odot (\widehat{\boldsymbol{b}} - \boldsymbol{b}))) \\ &+ (\Delta x \Delta y \Delta z \Delta t) J^T_{\widehat{\boldsymbol{b}}} (\widehat{\boldsymbol{R}} \odot \exp(\widehat{\boldsymbol{R}}) - \exp(\widehat{\boldsymbol{R}}) - \exp(\widehat{\boldsymbol{R}}) \log(|\boldsymbol{V}|) + |\boldsymbol{V}|) \\ &+ 2\beta(\Delta x \Delta y \Delta z)(J^m_{\widehat{\boldsymbol{b}}})^T (\widehat{\boldsymbol{\rho}}_{\boldsymbol{m}} - \boldsymbol{I}), \end{aligned} \quad \text{(Eqn-S19)}$$

$$\begin{aligned} g_{\widehat{\boldsymbol{R}}} = \frac{\partial \Gamma_s}{\partial \widehat{\boldsymbol{R}}} &= (\Delta x \Delta y \Delta z \Delta t) J^T_{\widehat{\boldsymbol{R}}} M((\widehat{\boldsymbol{b}} - \boldsymbol{b}) \odot (\widehat{\boldsymbol{b}} - \boldsymbol{b})) \\ &+ (\Delta x \Delta y \Delta z \Delta t)(J^T_{\widehat{\boldsymbol{R}}} (\widehat{\boldsymbol{R}} \odot \exp(\widehat{\boldsymbol{R}}) - \exp(\widehat{\boldsymbol{R}}) - \exp(\widehat{\boldsymbol{R}}) \log(|\boldsymbol{V}|) + |\boldsymbol{V}|) \\ &+ \mathrm{diag}(\exp(\widehat{\boldsymbol{R}}) \odot (\widehat{\boldsymbol{R}} - \log(|\boldsymbol{V}|))) \widehat{\boldsymbol{\rho}}) \\ &+ 2\beta(\Delta x \Delta y \Delta z)(J^m_{\widehat{\boldsymbol{R}}})^T (\widehat{\boldsymbol{\rho}}_{\boldsymbol{m}} - \boldsymbol{I}). \end{aligned} \quad \text{(Eqn-S20)}$$

For the Hessian matrix, we omit some complex second-order term and approximate it by

$$H = \Delta x \Delta y \Delta z \begin{bmatrix} h_{11} & h_{12} \\ h_{21} & h_{22} \end{bmatrix}, \quad \text{(Eqn-S21)}$$

where

$$\begin{aligned} h_{11} &= 2\Delta t \mathrm{diag}(\widehat{\boldsymbol{\rho}}^T M) + 2\beta(J^m_{\widehat{\boldsymbol{b}}})^T J^m_{\widehat{\boldsymbol{b}}} \\ h_{12} &= h_{21} = 2\beta(J^m_{\widehat{\boldsymbol{R}}})^T J^m_{\widehat{\boldsymbol{b}}} \\ h_{22} &= \Delta t \mathrm{diag}(\exp(\widehat{\boldsymbol{R}}) \odot (\widehat{\boldsymbol{R}} - \log(|\boldsymbol{V}|) + 1) \odot \widehat{\boldsymbol{\rho}}) + 2\beta(J^m_{\widehat{\boldsymbol{R}}})^T J^m_{\widehat{\boldsymbol{R}}}. \end{aligned}$$

1D simulations

We intend to use 1D simulations to illustrate what difference the prior information can make. The initial distribution ρ_0 and the final distribution ρ_1 are are described as follows:

$$\rho_0(x) = \begin{cases} \frac{3}{\sqrt{2\pi}\sigma_0} e^{-\frac{(x-\mu_1)^2}{2\sigma_0^2}} & 0 \le x \le 0.3, \\ \frac{10}{\sqrt{2\pi}\sigma_0} e^{-\frac{(x-\mu_2)^2}{2\sigma_0^2}} & 0.3 < x \le 1, \end{cases}$$

$$\rho_1(x) = \begin{cases} \frac{10}{\sqrt{2\pi}\sigma_0} e^{-\frac{(x-\mu_2)^2}{2\sigma_0^2}} & 0 \le x \le 0.7, \\ \frac{3}{\sqrt{2\pi}\sigma_0} e^{-\frac{(x-\mu_3)^2}{2\sigma_0^2}} & 0.7 < x \le 1, \end{cases}$$

where $\mu_1 = 0.2, \mu_2 = 0.5, \mu_3 = 0.8$, and $\sigma_0 = 0.1$. The initial and marginal distributions are shown in Extended Data Fig.**4a,b**. We first process the problem with gSB model without 'prior' information, i.e. $\widehat{b} = 0, V = 0$, and the interpolations are shown in Extended Data Fig.**4c** and Movie S1. In the interpolations, the left small Gaussian is merged into the center large Gaussian, and simultaneously, a small Gaussian is split out at the right and moves to the right. We then look at the case with influx/efflux prior. We use the prior with a little efflux at the left and a little influx at the right, and the interpolations are shown in Extended Data Fig.**4d** and Movie S2. In these interpolations, the left small Gaussian disappears gradually and there is a small Gaussian appears at the right. Finally, we tested it with a small velocity prior pointing towards the right, and the interpolations are shown in Extended Data Fig.**4e** and Movie S3. In this case, the left small Gaussian is moving to the right.

Parameter settings

We will explain all the parameters used in the results section in detail. The parameters include the model parameters in the formula (Eqn-S7) and discretization parameters like $\Delta t, m, \Delta x, \Delta y, \Delta z, n_x, n_y, n_z$.

In a 1D simulation, we focus on the spatial domain $[0, 1]$. The parameters are shown in Table 1.

In the lymph node, we used a constant prior V and tuned the value node by node. For each node, we performed gSB with $V = 0.5, 1, 1.5, 2$ and chose the one with the minimal maximum normalized square error between the uSB outputs and the raw Images. Specifically,

$$V = \underset{V=\{0.5,1,1.5,2\}}{\arg\min} \max_{0 \le j \le n-1} \frac{\|\widehat{\boldsymbol{\rho}}_{\boldsymbol{m}}^{\boldsymbol{j}} - I_{j+1}\|_2}{|I_{j+1}|_2} \qquad \text{(Eqn-S22)}$$

The χ will be determined manually on the summed image. Between two consecutive inputs, the parameters are shown in Table 2.

1.4 Comparison between interpolation and skipped data

We evaluated the gSB model by feeding it every second frame of the dataset, which resulted in one skipped frame between each consecutive pair. The model's interpolations were then compared to the original data. The time signal curves, presented in Supplementary Figure 6 A, show that the interpolation curve's overall trend aligns with the data. However, the interpolation also displays stair-like artifacts traced back to prior information from the first 10 minutes. A comparison of the signal maps from this initial period (Supplementary Figure 6 B) shows that the gSB-derived interpolations more effectively delineate the fluidic pathways of the medullary sinuses than the original data. This improved visualization of the pathways continues in later frames, as shown in the signal maps from the 80 to 90-minute interval (Supplementary Figure 6 C).

1.5 Sensitivity analysis

A sensitivity analysis was performed to evaluate the gSB model's dependence on denoising and segmentation parameters, focusing on the time-speed curve.

First, we investigated the model's sensitivity to the degree of denoising. As described in the Methods, our standard process involves 10 iterations of a 4D affine denoising filter. For this test, we reduced the process to 9 iterations and compared the outputs. We showed the comparison of the speed curve in Supplemental Figure 8A. The relative difference (mean±SD) between original denoising and less denoising in the speed curve is 0.0880 ± 0.0620.

Next, we examined the impact of segmentation. We translated the entry mask by a single voxel along the z-axis, either closer to or deeper into the dcLN. The original and translated masks maintained a high degree of similarity, with a Dice metric (mean±SD) of 0.64 ± 0.08. This small shift produced relative differences in the speed curve (0.0973 ± 0.0605), as seen in Supplemental Figure 8B.

1.6 LYVE-1 segmentation methodology

Data Preprocessing

Multi-channel images are denoted as

$$X = \{X_{\text{LYVE1}}, X_{\text{DAPI}}\}, \tag{Eqn-S23}$$

where X_{LYVE1} is the LYVE1 fluorescence channel and X_{DAPI} is the DAPI nuclear channel. Large images are divided into patches $\{X^{(k)}\}_{k=1}^{K}$ for processing.

In addition, X_{LYVE1} is binarized in Fiji (6) by manual thresholding to obtain $X_{\text{mask}}^{\text{LYVE1}}$. This binary mask is used both as ground truth for constructing paired datasets in U-Net training and as input for connected component analysis (CCA) in the connectivity branch (7).

Deep Learning Branch

We employed a U-Net predictor $\mathcal{U}$ (8) to segment LYVE1-positive sinus structures. The U-Net adopts a standard encoder–decoder design with skip connections, taking LYVE1 patches X_{LYVE1} as input and producing probability maps $\hat{Y}$ after a final sigmoid layer:

$$\hat{Y} = \sigma\big(\mathcal{U}(X_{\text{LYVE1}})\big). \tag{Eqn-S24}$$

Training is performed on paired data

$$\mathcal{D} = \{(X_i, Y_i)\}_{i=1}^{N}, \tag{Eqn-S25}$$

where X_i is a LYVE1 patch and Y_i the corresponding binary mask derived from Fiji preprocessing. Data augmentation follows common practice (9) and includes elastic deformation, random affine transformations, flipping, and brightness/contrast adjustment:

$$\mathcal{A}(X) = T_{\text{elastic}} \circ T_{\text{geo}} \circ T_{\text{flip}} \circ T_{\text{bc}}(X). \tag{Eqn-S26}$$

The model is optimized with BCEWithLogitsLoss:

$$\mathcal{L}(Y, \hat{Y}) = -\frac{1}{|Y|} \sum_{p \in Y} \big[Y(p) \log(\hat{Y}(p)) + (1 - Y(p)) \log(1 - \hat{Y}(p)) \big]. \tag{Eqn-S27}$$

Due to GPU memory limits, prediction on large images is performed in an overlapping patch-wise manner, and probabilities from overlapping regions are averaged before thresholding at 0.5 to obtain the final mask.

Postprocessing and contour filtering. After patch-wise prediction and probability averaging, we obtain a binary mask M_U by applying a threshold of 0.5. To refine this mask, we apply a contour filtering operator $\mathcal{F}(\cdot)$ that removes spurious or low-quality regions. The filtering criteria consider region size, proximity to image borders, mean DAPI intensity, and connectivity. Contours that do not satisfy these conditions are removed, yielding the refined U-Net prediction:

$$M_U^* = \mathcal{F}(M_U), \tag{Eqn-S28}$$

where $\mathcal{F}(\cdot)$ denotes the contour filtering algorithm based on connected component analysis and morphological constraints.

Connectivity Branch

This branch starts from the LYVE1-derived binary mask:

$$M_B^{(0)} = X_{\text{mask}}^{\text{LYVE1}}. \tag{Eqn-S29}$$

To recover sinus cavities that may be missed by the deep learning branch, we apply connected component analysis (CCA). CCA groups foreground pixels into maximally connected sets according to 8-connectivity, where pixels are considered neighbors if they share either an edge or a corner. Each connected component therefore represents a candidate cavity region. We denote the operation as

$$M_B^* = \mathcal{C}(M_B^{(0)}), \tag{Eqn-S30}$$

where $\mathcal{C}(\cdot)$ labels and extracts all connected regions. Based on the contour hierarchy, external contours correspond to cavity boundaries, while internal contours can be used to distinguish nested or spurious regions.

Fusion and Quantitative Analysis

The U-Net branch produces a refined mask M_U^* after contour filtering, while the connectivity branch produces a refined binary mask M_B^*. The two are combined by set union:

$$M_{\text{final}} = M_U^* \cup M_B^*, \tag{Eqn-S31}$$

where M_{final} denotes the complete sinus prediction mask.

To ensure predictions are restricted to valid tissue regions, we use a tissue mask M_T generated by EfficientSAM (10). The final valid mask is then obtained as

$$M_{\text{valid}} = M_{\text{final}} \cup M_T, \tag{Eqn-S32}$$

where $\cup$ denotes set union.

For quantitative analysis, let R be a detected sinus region containing n pixels. Given pixel resolution $\rho = 0.325\,\mu m$ and tissue slice thickness $t = 40\,\mu m$, the physical area and volume are computed as

$$\text{Area}(R) = n \cdot \rho^2, \qquad \text{Volume}(R) = \text{Area}(R) \cdot t. \tag{Eqn-S33}$$

Here Area(R) is the 2D area in μm^2, and Volume(R) is the corresponding 3D volume in μm^3.

Aggregating over all valid regions yields the total volume:

$$V_{\text{total}} = \sum_{R \in M_{\text{valid}}} \text{Volume}(R), \tag{Eqn-S34}$$

where V_{total} denotes the total tissue volume considered. Similarly, V_{LYVE1} represents the volume of LYVE1-positive regions and V_{sinus} the total sinus cavity volume. Their relative proportions are

$$P_{\text{LYVE1}} = \frac{V_{\text{LYVE1}}}{V_{\text{total}}} \times 100\%, \qquad P_{\text{sinus}} = \frac{V_{\text{sinus}}}{V_{\text{total}}} \times 100\%, \tag{Eqn-S35}$$

where P_{LYVE1} and P_{sinus} denote percentage contributions with respect to the total tissue volume.

Experimental Settings

Key parameters are summarized in Table 3.

2 Supplemental Figures

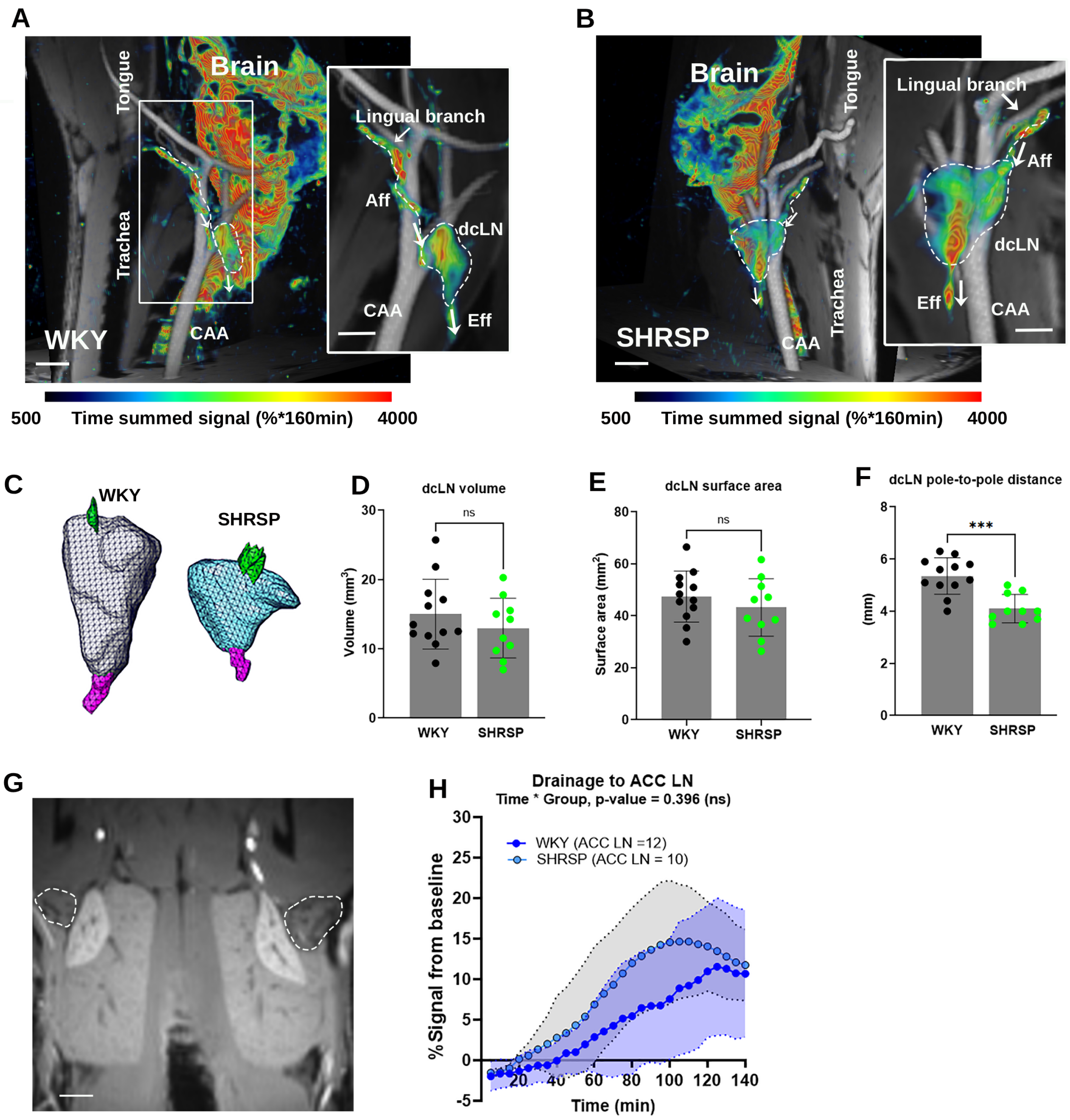

Supplemental Figure 1: **Drainage pathways and morphology of lymph nodes.** (**A**) 3D maximum intensity projection (MIP) summed DCE-MRI image of the brain, spine and drainage pathways to the deep cervical lymph nodes (dcLN) from a normal WKY rat. Landmarks are highlighted on the corresponding anatomical MRI 2D view planes. Dashed white lines highlight afferent lymphatic vessel and dcLN on the right side. (**B**) Corresponding data from a representative SHRSP rat highlighting the left dcLN. Aff=afferent lymphatic vessel; Eff=efferent lymphatic vessel; CAA=common carotid artery. Scale bars = 2mm. (**C**) 3D surface reconstructions of a WKY and SHRSP dcLN. The afferent and efferent vessels are highlighted in green and magenta colors, respectively. (**D-F**) Graphs with quantification dcLN volume (**D**), dcLN surface area (**E**) and pole-to-pole length of WKY (black circles) and SHRSP rats (green circles). Each dot above the bar represents values from one dcLN. Data are mean ± SD. Statistical analysis used a two-tailed independent t-test assuming unequal variances (Welch's t-test). ns = non-significant. ***p-value < 0.0001. (**G**) Anatomical MRI at the level of the submandibular gland. The dashed white outlines highlight the accessory lymph nodes. (**H**) Time signal curves (TSC) of drainage to the accessory lymph nodes observed in WKY (blue) and SHRSP (light blue) rats. Data are presented as mean and 95% confidence intervals. Statistical analysis used a linear mixed model for repeated measures with FDR correction for multiple comparisons. ns = non-significant.

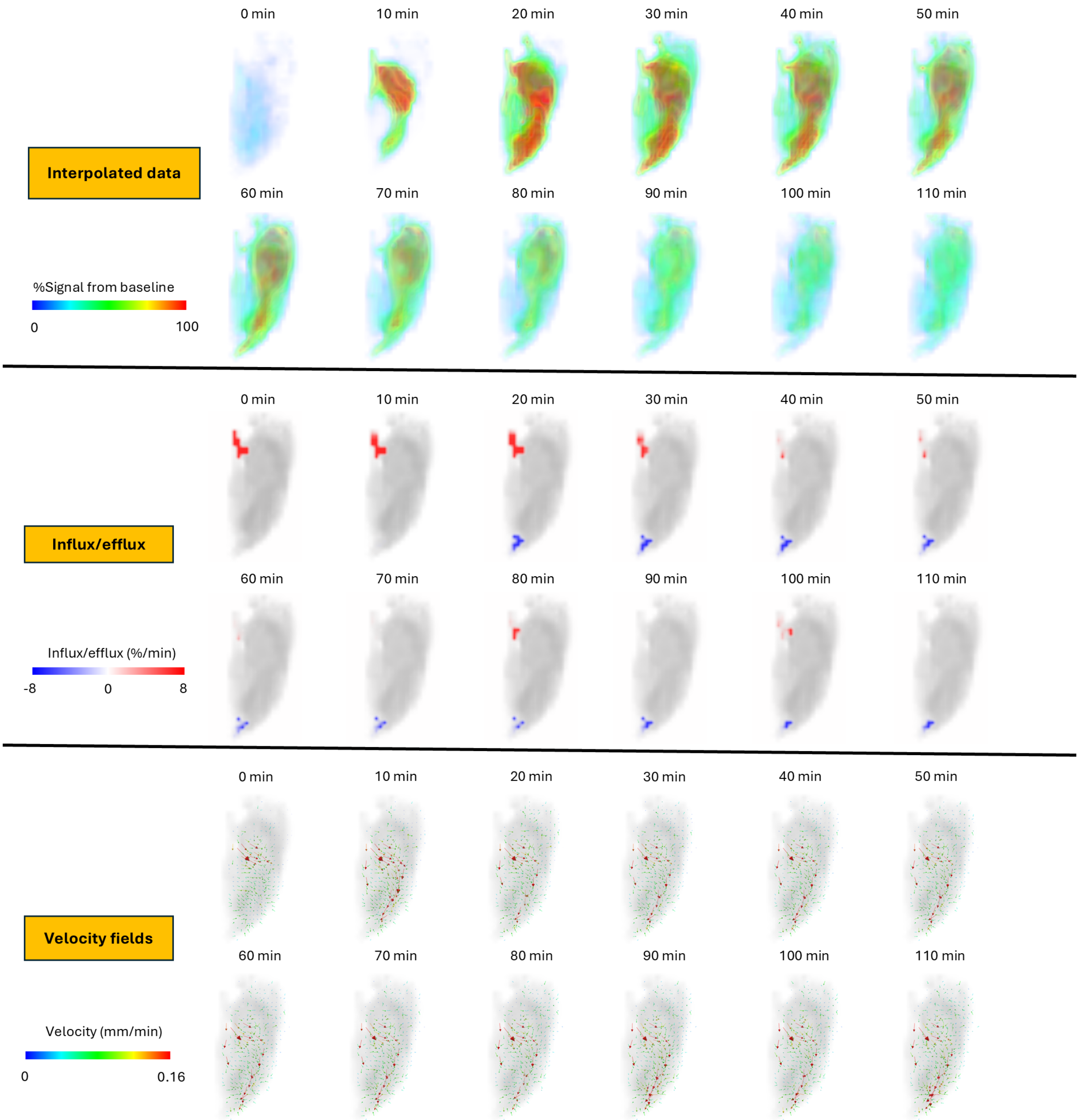

Supplemental Figure 2: Full time series of dcLN gSB outputs in Figure 2D. The time trajectory of interpolated data, influx/efflux map, and the velocity fields after the contrast solute's appearance in the node.

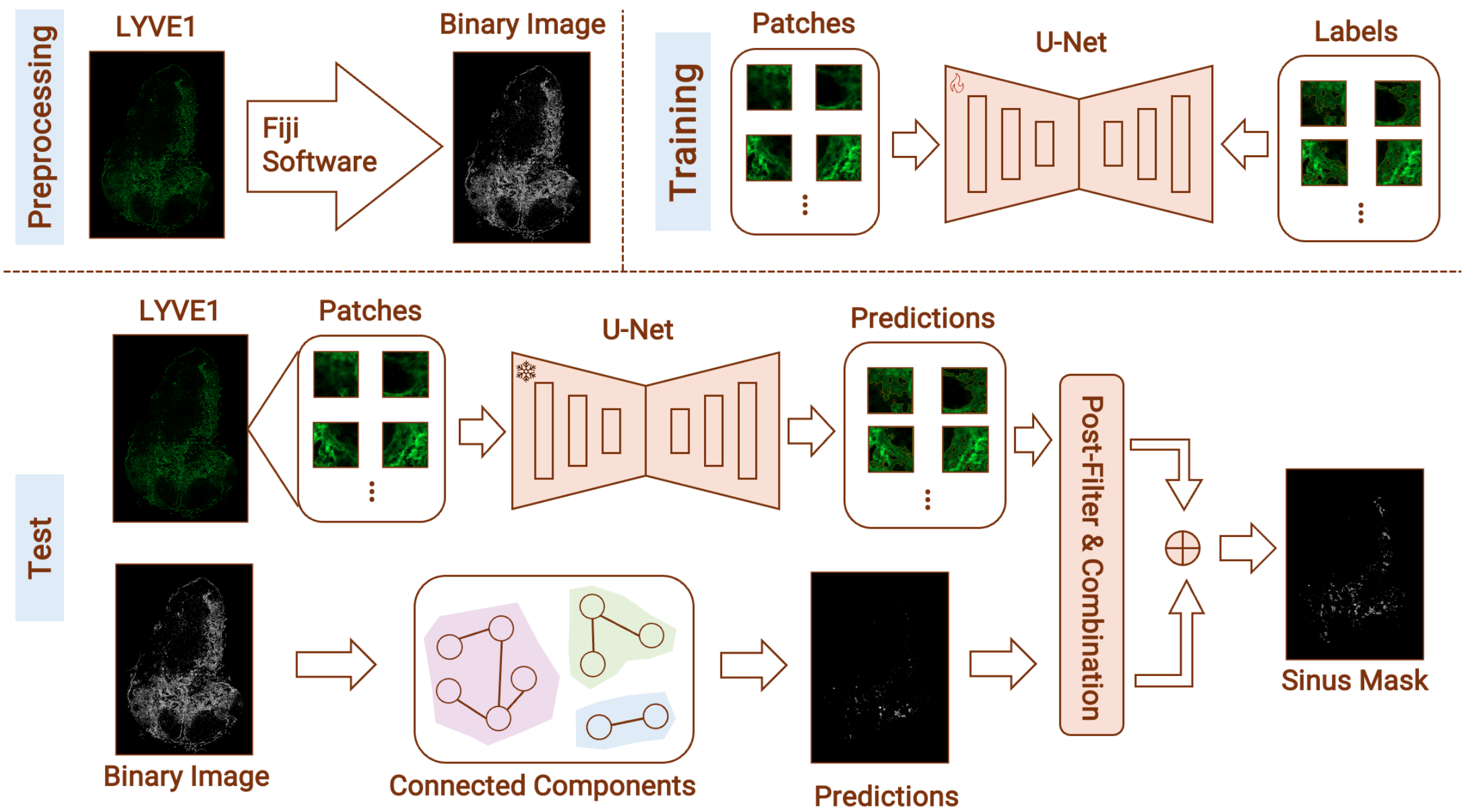

Supplemental Figure 3: **Pipeline of LYVE-1 image processing. Step 1:** Preprocessing in Fiji to generate a LYVE1-derived binary mask and extract patches. **Step 2:** Deep learning branch uses U-Net for patch-wise prediction of LYVE1-positive structures. **Step 3:** Connectivity Branch applies connected component analysis (CCA) on the LYVE1- derived binary mask. **Step 4:** Fusion and quantitative analysis combine both outputs, filter with tissue masks, and compute sinus morphology statistics.

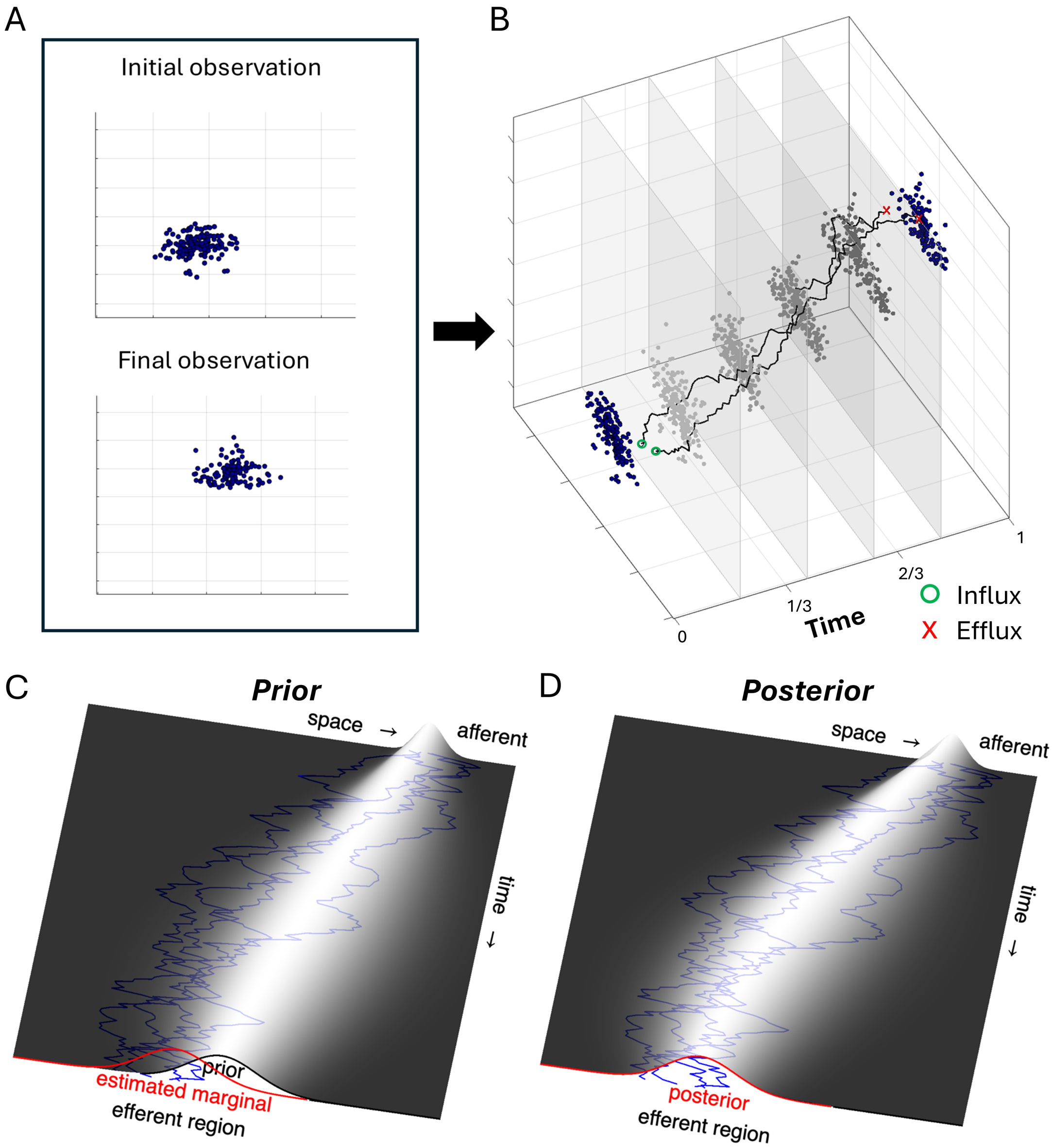

Supplemental Figure 4: **gSB illustration:** (**A** and **B**) Exemplification of data and output of the gSB model – initial and final point clouds are reconciled with available prior to provide the most likely flowlines as output. (**C** and **D**) Schematic of advective (directional) and diffusive (random) solute transport in one spatial dimension, with particles entering in the afferent and exiting in the efferent regions – a comparison of spatio-temporal distributions (envelops) of prior (**C**) and posterior (**D**) dynamics is shown. Representative solute trajectories are drawn in blue.

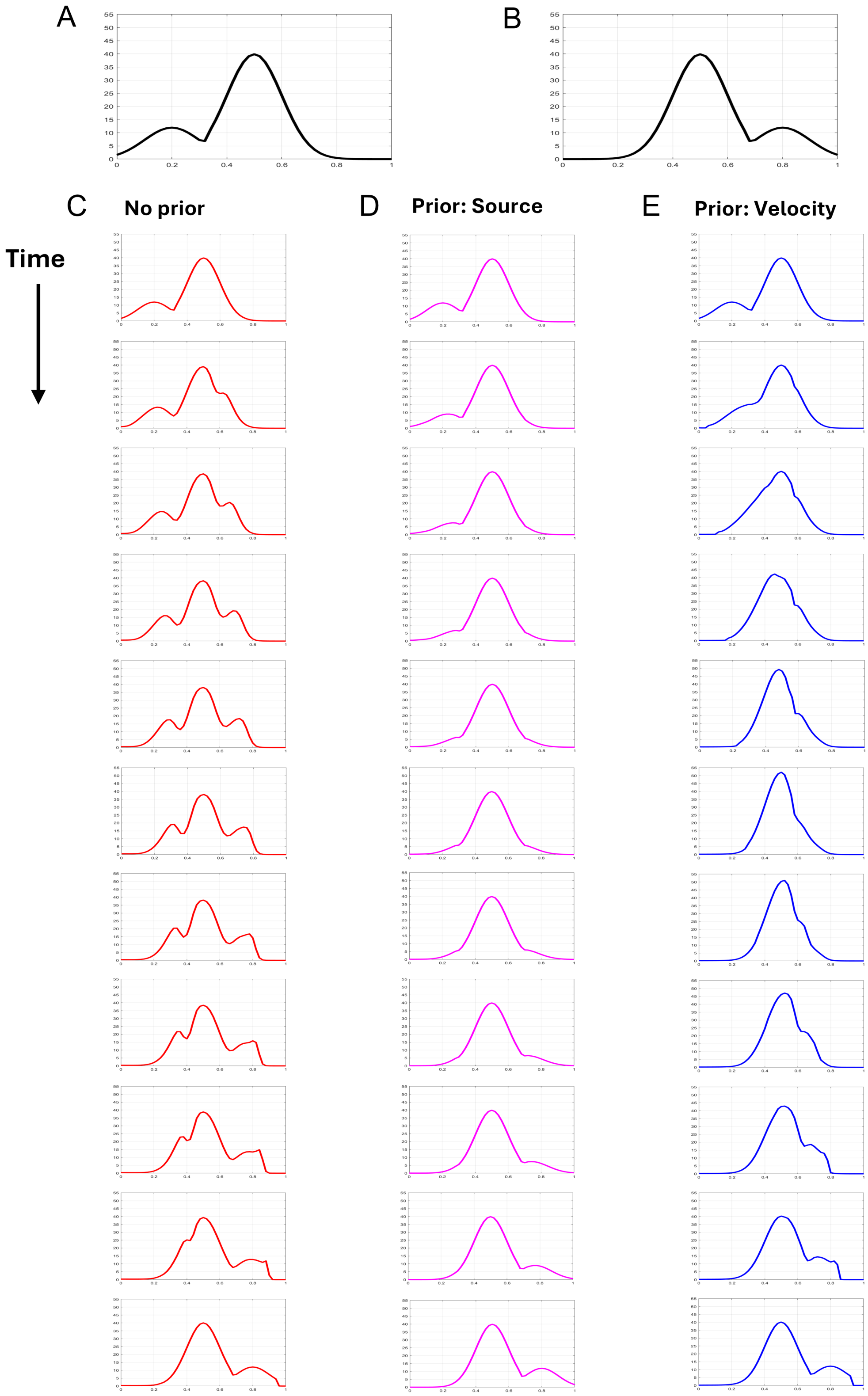

Supplemental Figure 5: **1D simulation.** (**A** and **B**) Initial and final marginal distributions. (**C**) Interpolation without prior. (**D**) Interpolation with prior on influx/efflux rates. (**E**) Interpolation with prior having a drift (velocity) term.

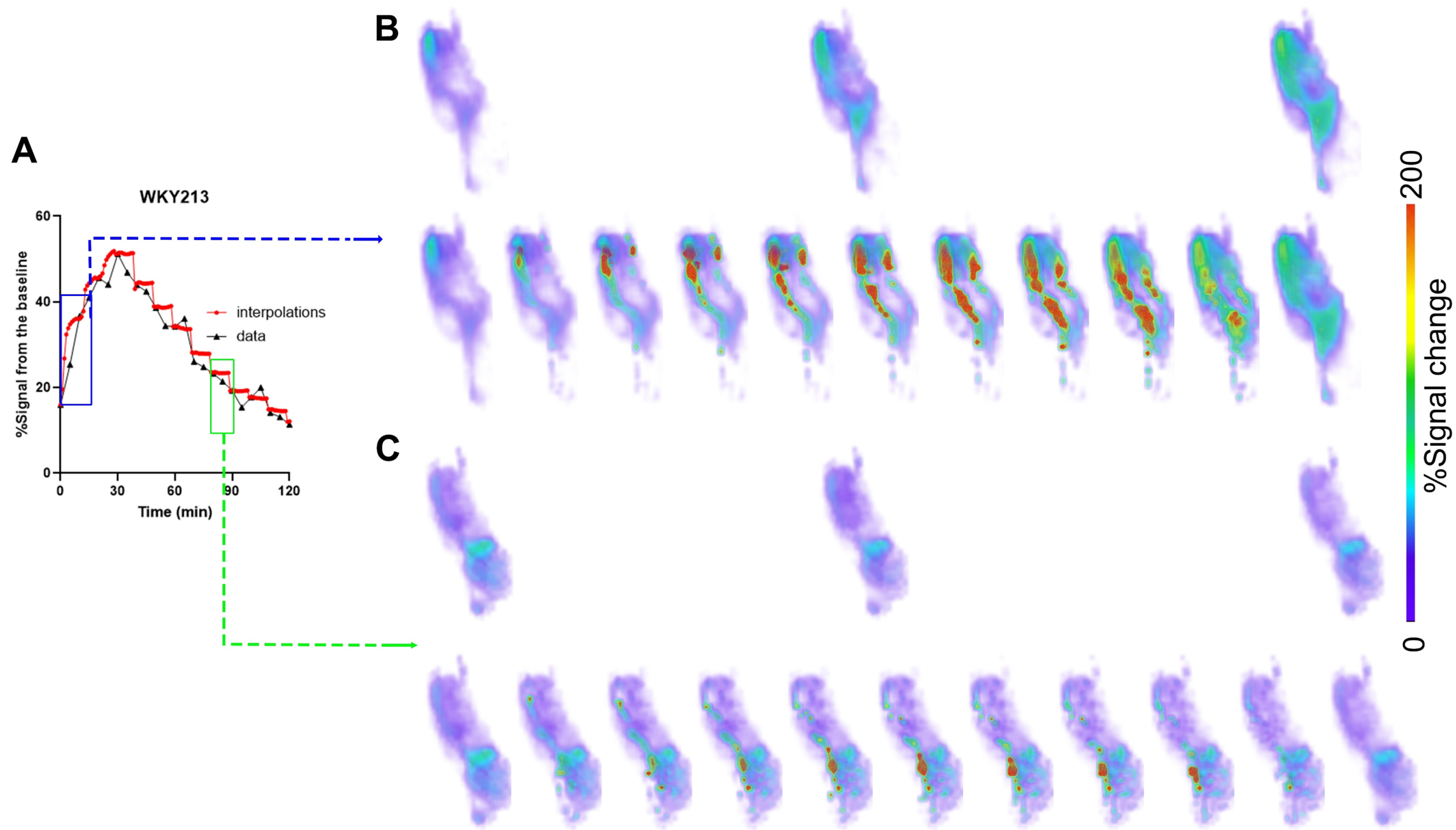

Supplemental Figure 6: **Comparison between interpolations and skipped data.** (**A**) Time signal curve of a WKY rat. The black curve is the preprocessed data and the red one is the interpolation derived from the gSB model. (**B**) Signal change map of data and gSB-derived interpolation from 0 to 10 minutes. (**C**) Signal change map of data and gSB-derived interpolation from 80 to 90 minutes.

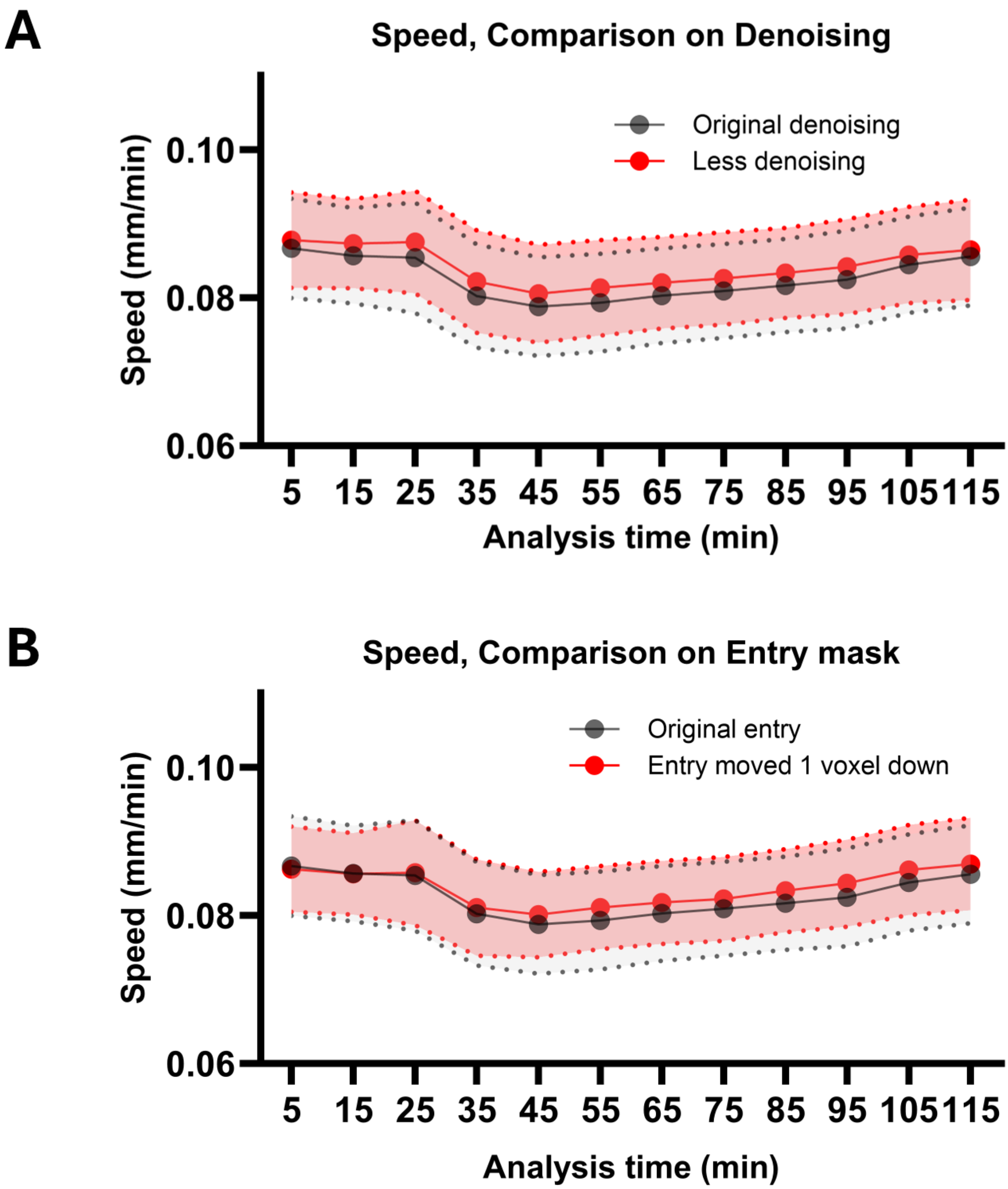

Supplemental Figure 7: **Sensitivity analysis** (**A**) Comparison of time-speed curves across different denoising settings. (**B**) Comparison of time-speed curves across different entry mask settings. Data is presented as mean with 95% confidence intervals

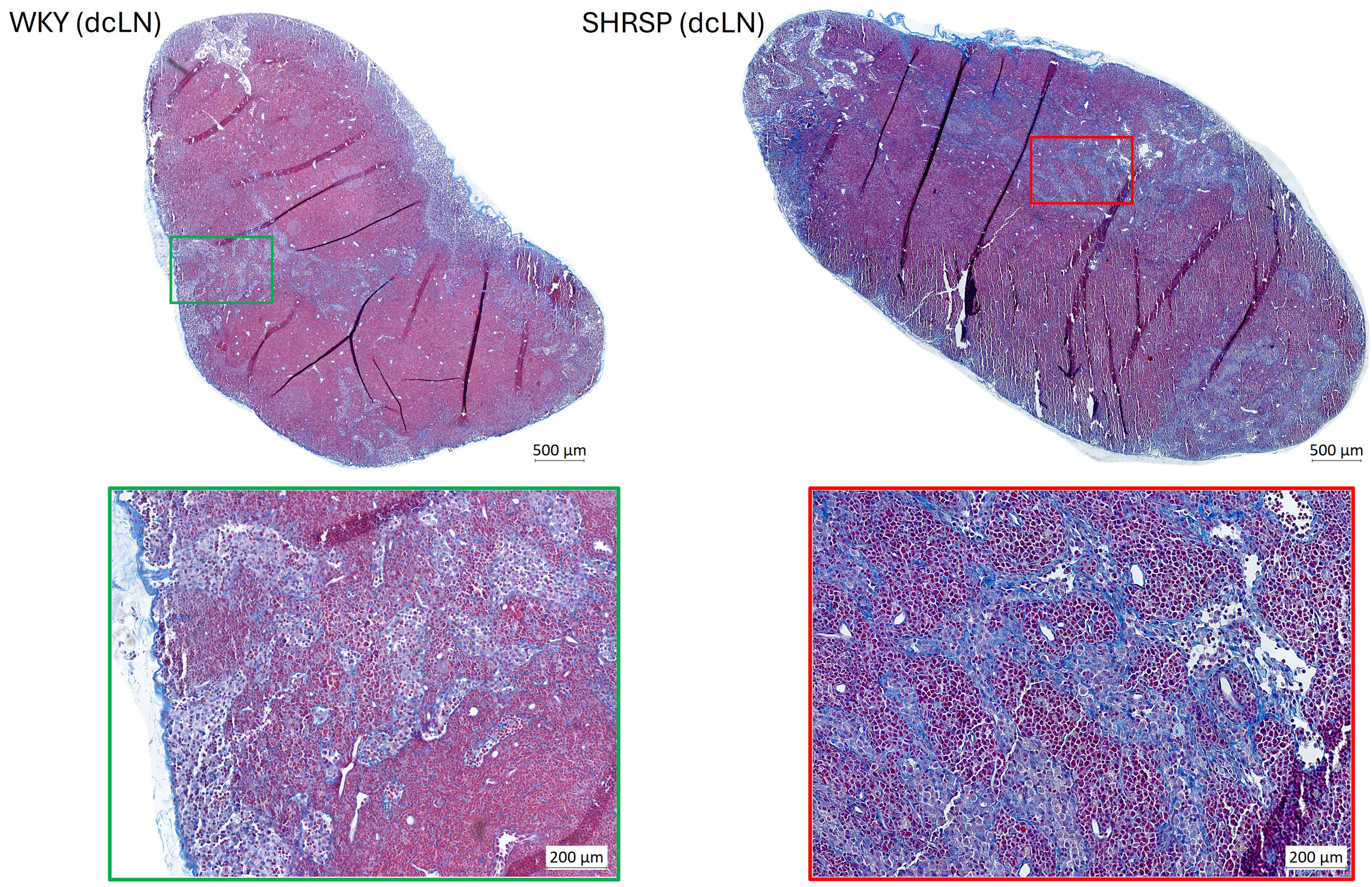

Supplemental Figure 8: Representative trichrome-stained sections from one WKY and one SHRSP deep cervical lymph node, with zoomed-in views of the collagen-rich regions associated with the sinuses.

3 Supplemental Tables

3.1 Table S1: Parameters table of 1D simulation

n_x	50
Δx	0.02
m	30
Δt	1/30
D	0.05
β	1000000
V_0	Depend on prior cases, details in codes
χ	Depend on prior cases, details in codes
b	Depend on prior cases, details in codes

Table 1: Parameters of 1D simulation

3.2 Table S2: Parameters table of Lymph node study

n_x, n_y, n_z	determined by dcLN mask
Δx, Δy, Δz	1,1,1
m	10
Δt	0.4
D	0.002
β	10000
V_0	tuned from {0.5, 1, 1.5, 2}
χ	$\begin{cases} 1 & \vec{x} \in \text{influx region} \\ -1 & \vec{x} \in \text{efflux region} \\ 0 & \text{o.w.} \end{cases}$
b	0 first frame; velocity from the previous frame

Table 2: Parameters of Lymph node study

3.3 Table S3: Summary of LYVE-1 Segmentation Parameters

Category	Parameters
Dataset Construction	Non-overlapping patches; poor-quality masks excluded
Data Augmentation	Elastic deformation (p=0.2); Geometric transforms (rotation, scale, translation, p=0.5); Flipping (p=0.5); Brightness/contrast adjustment (p=0.2) (9)
Training Config	Learning rate 1×10^{-4}; 100 epochs; Batch size 64; Optimizer: Adam; Loss: BCEWithLogitsLoss; GPU: RTX3090; Train/Val split 80/20
Model Architecture	Standard U-Net with encoder–decoder design and skip connections; final 1×1 convolution + sigmoid output
Inference Params	Patch size 256×256; Stride 32; Overlap 87.5%; Threshold 0.5 (sigmoid)
Contour Filtering	Filtering by region size, border proximity, mean DAPI intensity, and connectivity
Connectivity Branch	LYVE1-derived binary mask from Fiji; 8-connectivity CCA; contour hierarchy separation
Fusion	Union of deep learning and connectivity outputs; refinement with EfficientSAM tissue mask
Physical Params	Resolution 0.325 μm; Slice thickness 40 μm

Table 3: Summary of LYVE-1 Segmentation Parameters

References

1. Chen Y, Georgiou TT, and Pavon M. Stochastic control liaisons: Richard Sinkhorn meets Gaspard Monge on a Schrödinger bridge. SIAM Review 2021; 63:249–313
2. Chen Y, Georgiou TT, and Pavon M. On the relation between optimal transport and Schrödinger bridges: A stochastic control viewpoint. Journal of Optimization Theory and Applications 2016; 169:671–91
3. Chen Y, Georgiou TT, and Pavon M. The most likely evolution of diffusing and vanishing particles: Schrodinger bridges with unbalanced marginals. SIAM Journal on Control and Optimization 2022; 60:2016–39
4. Chen Y, Georgiou TT, and Pavon M. Optimal Survival Strategies for Diffusive Flows: A Schrödinger Bridge Approach to Unbalanced Transport. SIAM Review 2025; 67:579–604
5. Steklova K and Haber E. Joint hydrogeophysical inversion: state estimation for seawater intrusion models in 3D. Computational Geosciences 2017; 21:75–94
6. Schindelin J, Arganda-Carreras I, Frise E, Kaynig V, Longair M, Pietzsch T, Preibisch S, Rueden C, Saalfeld S, Schmid B, et al. Fiji: an open-source platform for biological-image analysis. Nature methods 2012; 9:676–82
7. Jähne B. Digital image processing. Springer, 2005
8. Ronneberger O, Fischer P, and Brox T. U-net: Convolutional networks for biomedical image segmentation. *International Conference on Medical image computing and computer-assisted intervention.* Springer. 2015 :234–41

9. Wang J, Perez L, et al. The effectiveness of data augmentation in image classification using deep learning. Convolutional Neural Networks Vis. Recognit 2017; 11:1–8

10. Xiong Y, Varadarajan B, Wu L, Xiang X, Xiao F, Zhu C, Dai X, Wang D, Sun F, Iandola F, et al. Efficientsam: Leveraged masked image pretraining for efficient segment anything. *Proceedings of the IEEE/CVF Conference on Computer Vision and Pattern Recognition.* 2024 :16111–21
